# Characterization of recombinase-based genetic parts and circuits using nanopore sequencing

**DOI:** 10.1038/s41467-026-77605-x

**Published:** 2026-09-18

**Authors:** F. Veronica Greco, Sarah K. Cameron, Shivang Hina-Nilesh Joshi, Sarah Guiziou, Jennifer A. N. Brophy, Claire S. Grierson, Thomas E. Gorochowski

**Affiliations:** 1 School of Biological Sciences, University of Bristol, Bristol, UK; 2 Earlham Institute, Norwich Research Park, Norwich, UK; 3 Department of Bioengineering, Stanford University, Stanford, CA USA; 4 Howard Hughes Medical Institute, Stanford, CA USA; 5 Present Address: CDotBio Ltd, Future Space, Bristol, UK; 6 Present Address: Department of Life Sciences, University of Bath, Bath, UK

**Keywords:** Synthetic biology, DNA sequencing, Biotechnology, Genetics

## Abstract

Recombinases are versatile enzymes able to perform the precise insertion, deletion, and rearrangement of DNA and can act as a foundation for programmable genetic logic and memory. Crucial for their use are accurate measurements of function. However, these are often laborious, time-consuming, and costly to collect. To address this, we develop a semi-automated workflow that combines low-cost liquid handling robotics, multiplexed long-read nanopore sequencing, and a supporting computational analysis tool to enable the high-throughput and detailed characterization of recombinase parts and circuits when used in a variety of contexts and organisms. Our approach overcomes the limitations of typically used fluorescence-based assays and is able to monitor temporal dynamics, observe structural changes at nucleotide resolution, and unravel the internal workings of complex multi-state circuits. The ability to scale up and automate genetic circuit characterization is an essential step towards more rigorous biological metrology that can support the construction of predictive models for efficiently engineering biology.

## Abstract (web summary)

Measuring the accuracy of recombinase activity is often laborious, time-consuming, and costly. Here, the authors develop a semi-automated workflow that enables the high-throughput characterization of recombinase parts and circuits in a variety of contexts and organisms.

## Introduction

The ability to reprogram cellular behaviors through the engineering of biology has the potential to address global challenges spanning sustainability to healthcare^1^. Synthetic genetic circuits offer a way of tapping into the computational capabilities of cells^2^, rewiring and extending their functionalities in new ways^3,4^. To date, most genetic circuits have been built using protein-based transcription factor networks. These offer a flexible platform for regulating the flows of transcription within a cell and allow for small regulatory motifs encoding basic computational operations (e.g., logic gates) to be easily combined to create larger circuits implementing more complex computations^5^. While these circuits have been used to implement diverse information processing in a wide range of organisms^5–12^, they require the precise and continuous expression of circuit components, which can be affected by stochastic molecular fluctuations and circuit context (genetic, host and environment)^13^, and they impart a continuous burden on the cell^14^. This can lead to circuits whose functions are not always reliable and which are difficult to scale in complexity^5,15–17^ without introducing advanced self-regulation^18^ or the ability to tune functionalities after assembly^19,20^.

In contrast, recombinase-based circuits encode their state directly within DNA, using enzymes like integrases to cause modifications through the addition, removal, and rearrangement of DNA sequences^12,21,22^. This allows for the implementation of robust computational operations that are discrete in nature (i.e., a DNA sequence is either present or absent and has a specific orientation). Recombinase-based circuits have been used to create robust genetic logic^8–12,23–26^ and memory arrays that encode ‘bits’ of information in the orientation of short independent segments of DNA^21^. Such circuits hold much promise, but their development is hampered by a limited number of well-characterized recombinase enzymes^27^ and difficulties debugging large non-functional circuits. This stems from the fact that typical monitoring tools like fluorescent proteins are only able to simultaneously capture a handful of internal circuit states^28^, making it impossible to understand how a large multi-state circuit is actually functioning. Furthermore, the expression of fluorescent reporter proteins often requires changes to be made to a circuit. These changes can themselves alter the function and introduce delays in measurements, complicating the characterization of circuit dynamics. New experimental methods are needed that enable the full state of these systems to be monitored more directly over time. Long-read sequencing could hold the answer, but its high cost and typically low-throughput nature have so far limited its use in synthetic biology^29,30^.

Here, we address this issue by developing a semi-automated workflow to enable the high-throughput and detailed characterization of large recombinase-based genetic circuits. We show how low-cost liquid handling robotics can be combined with nanopore sequencing and computational analysis tools to monitor the complete state of recombinase circuits over time. We demonstrate our workflow’s capabilities by simultaneously characterizing the kinetics and off-target activity of six recombinase enzymes, monitoring the dynamics of a multi-plasmid recombinase-based genetic cascade, and showing how spatial activation of recombinase switches can be quantified across plant roots. The ability of our workflow to process up to 96 samples in 2 days using a single Opentrons OT-2 robot ensures that it is accessible to most labs, supporting the roll-out of more comprehensive metrology for engineering biology.

## Results

### A workflow for characterizing recombinase-based parts and circuits

**Fig. 1:**
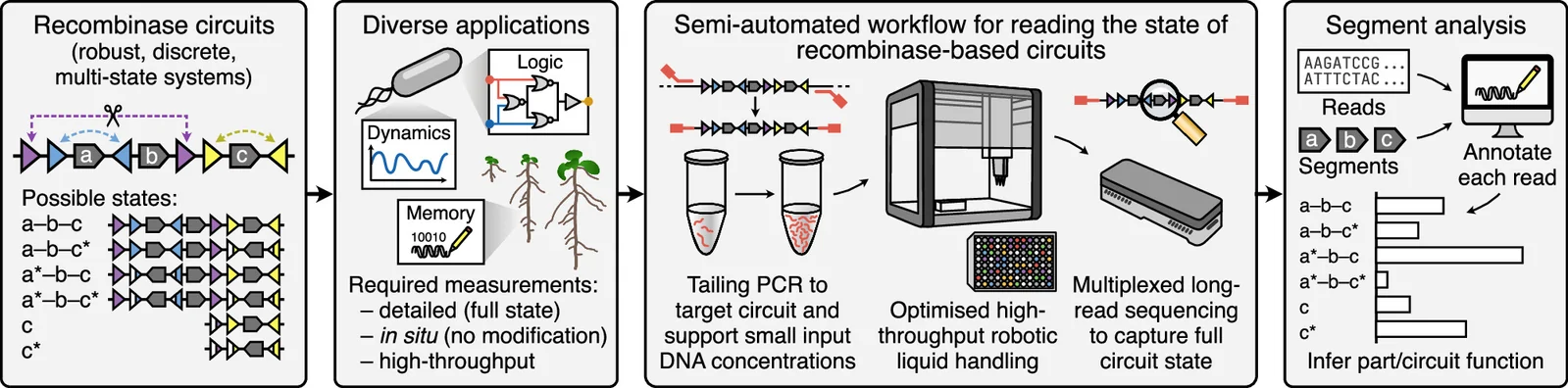
Overview of the characterization workflow. (left–right) Recombinase circuits enable the creation of robust multi-state systems for biological computation and memory. Our workflow allows for small DNA samples to be taken and uses PCR to ‘tail’ a target region containing the circuit for analysis via long-read nanopore sequencing. The tailing PCR and subsequent sequencing library preparation can be carried out in high-throughput using optimized protocols on a low-cost liquid handling robot (Opentrons OT-2). These libraries are then nanopore-sequenced to provide full-length information about the circuit state. Finally, analysis of the sequencing data is performed using segment analysis, where user-provided segments (DNA sequences) that can be used to capture the full state of the circuit are searched for within each read and for all samples processed. The ‘segment’ computational tool automates this step, outputting the presence, order, and orientation of each provided segment within each read. Distributions of the states present in a sample can then be used to characterize circuit function and dynamics for samples taken over time.

Our workflow consists of four steps that cover DNA extraction, target amplification, sequencing, and analysis (Fig. 1 and Supplementary Notes 1 and 2 and Supplementary Tables 1 and 2). DNA extraction depends specifically on the application and type of organism, but can be performed using standard methods like heat, chemical or mechanical lysis. After DNA extraction is complete, we then provide automated liquid handling protocols for Opentrons OT-2 robots based on a standard 96-well plate format, which can also be performed manually if required (Supplementary Note 3; Supplementary Data 1). The workflow starts by amplifying the recombinase circuit via a ‘tailing’ PCR. This PCR uses primers designed to target the start and end of the recombinase circuit and append common DNA sequences to both ends of the amplicons to aid later barcoding. Once these PCR reactions are complete, they are separately purified using magnetic beads and the DNA concentration for each sample quantified. These DNA quantities are fed back into the workflow to allow automated normalization of concentrations before a further barcoding PCR step is performed. This PCR makes use of primers that target the common tails added to each amplicon and further appends a unique barcode sequence to allow for distinguishing the source sample after sequencing. These sample-specific PCR reactions are then separately purified using magnetic beads and their DNA is quantified before pooling in equimolar amounts. This single pooled sample is then sequenced after a standard ligation-based nanopore sequencing library preparation.

During the development of the experimental protocols, several optimizations were made to reduce sequencing costs and handling time, while maintaining data quality and quantity. Some of the key findings were that: (i) sample volumes as small as 25 μL from bacterial liquid cultures provided enough template material for amplification, (ii) the use of a heat-lysis step after flash freezing in liquid nitrogen was sufficient to extract DNA for both bacteria and dissected plant tissues, and (iii) magnetic bead purification could support size selection of target amplicons such that non-ligated sequencing adapters were efficiently removed (Supplementary Fig. 1), improving overall sequencing throughput.

Once sequencing is complete, analysis of the sequencing data is carried out using our computational tool called ‘segment’ (*Methods*; Supplementary Fig. 2; Supplementary Data 2). Because the state-space of a recombinase circuit grows exponentially as new recombination events become possible (i.e., the addition of a new set of recombinase sites can potentially triple the size of the state space through the absence or presence of a new region of DNA in either a forward or reverse direction), we do not align reads to all possible reference sequences. Instead, the segment tool takes as input short ‘segment sequences’ in a multi-FASTA format. Segment sequences need to be provided for the start and end of the circuit, as well as any regions within the circuit that undergo change (e.g., addition/removal or a change in orientation) as the circuit functions. By focusing on the segments that change rather than every possible state of the full circuit, much less information needs to be provided by a user. Furthermore, the information given allows for every read to be fully classified by searching for the start and end segments to orientate the sequence, and then searching for all other possible segments to generate a full classification that is encoded as a concatenation of the segment names in the order they are found from the start to end segment, with the orientation of each segment being denoted by the absence or presence of an asterisk appended to the segment name (e.g., if segment S1 is found in a reverse orientation then it is denoted by S1* in the read classification). Using this approach, all reads can be classified and a summary of observed states and their frequency generated. From these summaries, it is then possible to assess the overall circuit function (Fig. 1).

### Detailed characterization of a recombinase library

**Fig. 2:**
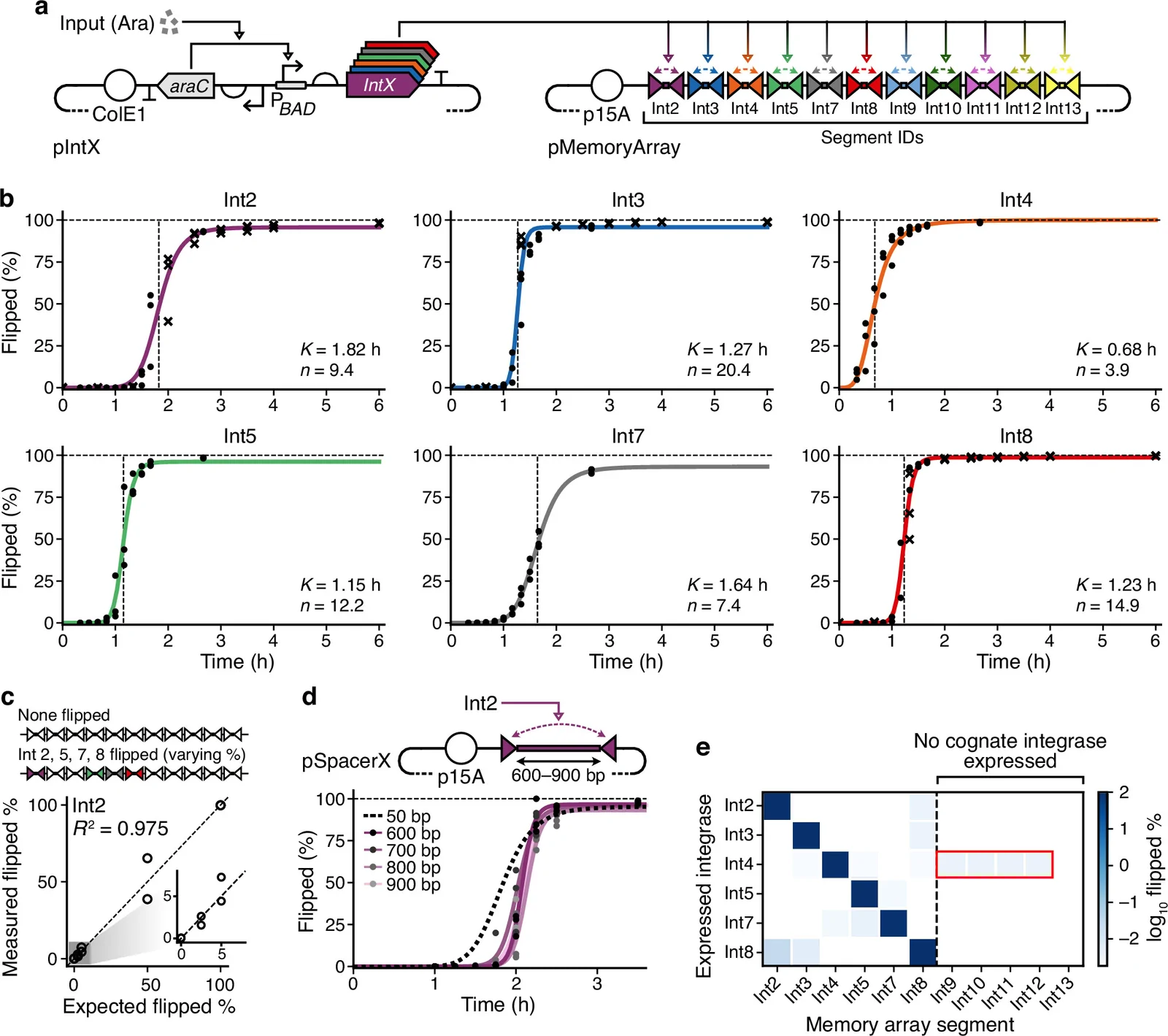
Detailed characterization of six recombinases. **a** Schematic of the integrase expression plasmids (pIntX, left) and the memory array (pMemoryArray, right) that contains 11 different integrase recognition site pairs (*attB* and *attP*) flanking short DNA sequences such that cognate integrase expression causes a non-reversible inversion (flip) of that specific segment of DNA, permanently recording an event. **b** Characterization of the temporal activity of 6 integrase enzymes. Solid lines show a fitted Hill function with *K* and *n* parameters shown in the bottom right corner of each plot. *K* value is also denoted by a vertical dashed line. Black circles and crosses are individual measurements from the three biological replicates for samples taken at differing time points: 0, 40, 80, 120, 150, 180, 210, 240, and 360 min, and 20, 30, 40, 50, 60, 70, 80, 90, 100, and 160 min, respectively. **c** Characterization of populations of cells with known mixed proportions of memory array plasmids with Int2, 5, 7, and 8 flipped. Data shown for Int2 with samples containing 0%, 2.5%, 5%, 50% and 100% flipped states. Average of duplicate experiments shown by unfilled circles and error bars denote the standard deviation. Inset shows a zoomed-in region near the origin and *x* = *y* is shown by the dashed line. **d** Testing the effect of different lengths of DNA (600–900 bp) between recognition sites of the Int2 integrase on the temporal flipping of the target site (solid colored lines). Fit for 50 bp spacer from panel ‘b’ shown as a dotted black line. Schematic of the pSpacerX plasmids built for this experiment shown right of the plot. Solid circles are individual measurements from the three biological replicates for samples taken at differing time points. **e** Orthogonality matrix for the 6 integrases tested and 11 integrase recognition sites for samples taken at 2 h 40 min. Off-target activity is denoted by the flipping of non-cognate recognition sites. Red box highlights off-target activity that affects four sites (Int9 to Int12) that are not targets for any of the integrases expressed in the experiment. Source data for this figure is available in the Source Data file.

To demonstrate the core features of our workflow, we performed a detailed characterization of six large-serine recombinases (Int2, Int3, Int4, Int5, Int7, and Int8) in *Escherichia coli* that were previously mined from sequence databases^21,27^. First, we assessed the dynamic response of the recombinases by monitoring the inversion (flipping) of their cognate recognition sites over time after recombinase expression was induced. We made use of a previously assembled 11-bit memory array that consisted of 11 short DNA sequences where each was flanked by a specific pair of recombinase recognition sites in orientations that would cause an inversion in the DNA sequence between them when the cognate recombinase is present (Fig. 2a). The orientation of these 11 short sequences can capture up to 2^11^ = 2048 different states.

Cells were co-transformed with plasmids allowing for induction of a specific recombinase and containing the 11-bit memory array. Experiments were then performed where recombinase expression was induced (at *t* = 0 h) and then samples were taken over a 6-h period for processing by our workflow (Fig. 2b, black filled circles). To analyze the state of the memory array, we provided sequences for each of the flanked regions as segments (labeled ‘IntX’, where ‘X’ is the recombinase of interest) and calculated the percentage that had flipped by comparing the IntX (unflipped) to IntX* (flipped) states for each recombinase in each read using the segment tool. After this initial experiment, we found that for some of the recombinases, the complete response curve was not well captured. We therefore performed an additional experiment with samples taken over a shorter 2 h 40 min period for a subset of the recombinases (Fig. 2b, black crosses). Combining data from both these experiments, we were able to recover complete response curves for all six recombinases. Even though the response of many of the recombinases was fast, taking less than 30 min to transition to 100% flipped (e.g., for Int3 and Int8), we saw fairly similar responses across measurements from biological replicates, demonstrating good reproducibility in the method. Hill function fits to the data for strong expression of the recombinases showed a large diversity in response dynamics, with the time to reach 50% of cells having flipped DNA (*K* values) ranging from approximately 40 to 110 min and cooperativities (*n* values) spanning from 3.9 to 20.4 (Fig. 2bb and Supplementary Table 3).

To assess the accuracy of measurements from the workflow, we performed further experiments with populations of cells containing known fractions of the memory array where a specific set of sites had been flipped. To do this, we generated memory array plasmids in two different states. The first state was the original plasmid with all sites unflipped. The second state was the memory array after exposure to Int2, Int5, Int7 and Int8 such that the cognate sites for those recombinases had become fully flipped. Plasmids in this second state were then purified and sequence verified to ensure they contained the correct configuration. Finally, two separate populations of cells were transformed: the first with the original unflipped memory array, and the second with the purified, semi-flipped memory array. Importantly, cells from each population contained no recombinase expressing plasmid (i.e., pIntX), ensuring no further changes would be made to the state of the memory array. These two populations were then separately grown, their optical density at 600 nm (OD600) measured and varying proportions of the populations mixed to generate expected fractions of unflipped and flipped memory arrays. We chose mixtures containing 0%, 2.5%, 5%, 50% and 100% of flipped cells to be able to assess the sensitivity of the workflow to small differences, while also testing the full range of possible values. Comparing the expected flipped percentage of the Int2, Int5, Int7 and Int8 sites to those measured, we saw a strong correlation with *R*^2^ > 0.975 for each site (Fig. 2c). Also, as expected, virtually no flipping of other recombinase sites was seen, with only two samples showing very low levels of incorrect flipped states for three of the recombinase sites (<0.002% for Int3, Int4 and Int12). These results suggest that the workflow is accurate and sensitive enough to measure even small changes (<10%) in the state of recombinase sites within the memory array.

It is known that the genetic context (i.e., nearby flanking sequences and distances between recombination sites) can affect the function of recombinases^31,32^. To assess this aspect, we carried out a complementary experiment for Int2 to explore whether the length of DNA flanked by recombinase sites affected the efficiency and dynamics of flipping. For lengths from 600 to 900 bp (typical for a protein-coding gene), we saw no substantial difference (Fig. 2d and Supplementary Table 3). However, when comparing the rates of flipping (i.e., steepness of the response function) of 600–900 bp segments to the short 50 bp segment of the memory array, there was a notable decrease with *n* values shifting from ~21 to 9.4 for the short segment (Fig. 2d, dotted line). This could stem from physical constraints in the accessibility and bending of DNA that are required for efficient recombination. It also suggests that the design of circuits using recombinases may need to consider the lengths of flanked DNA regions to ensure a desired function is achieved.

For multiple recombinases to be used concurrently within a circuit, it is essential that they display minimal off-target activities. Typically, when using fluorescence reporters to monitor recombination events, only a small number of alternative sites can be simultaneously tested due to the spectral overlap of the reporter proteins. Our methodology is not limited by this constraint. Because we use an array of recombinase recognition sites that can be read in its entirety by a single sequencing read, we are able to simultaneously measure the off-target activity at all 11 recombinase sites. This substantially simplifies the characterization of off-target effects and could easily be scaled to much larger numbers of potential sites as long-read sequencing has been shown to produce reads more than 100 kb in length.

More detailed analysis of the sequencing data from the memory array showed that all recombinases precisely targeted their cognate sites, with only minor off-target activity for Int4 across numerous recognition sites (Int9–Int12), Int2–Int4 weakly recognizing Int8 sites, and some cross-reactivity between Int5 and Int7, Int2 and Int8, and Int3 and Int8. In all cases, off-target activity was typically very rare with <0.1% of off-target regions flipped after 2 h 40 min (Fig. 2e). This off-target activity relates to <10 reads per interaction and in most cases was no more than 1 or 2 reads across multiple biological replicates. It is possible that some of the off-target activity occurred due to incorrect barcode classification during the sequencing step^33^. For example, a read from a sample where Int8 is expressed might be incorrectly classified as a read for a sample where Int2 is expressed. In this scenario, given the rarity of barcode misclassifications, we would expect only the off-target site to be flipped. Further analysis of all reads containing off-target activity showed this to be the case, with 83% of a total of 29 reads showing off-target activity across all integrases being potentially prone to barcode misclassification. However, we still found 2 reads in which both the correct site was flipped in addition to an off-target site where barcode misclassification is unlikely to have played a role, and this possible error could not account for the additional 3 reads across two biological replicates showing off-target activity for the entire Int9–Int12 region when expressing Int4. For this particular case, none of the flipped off-target sites had their cognate integrase expressed in any of the samples.

**Fig. 3:**
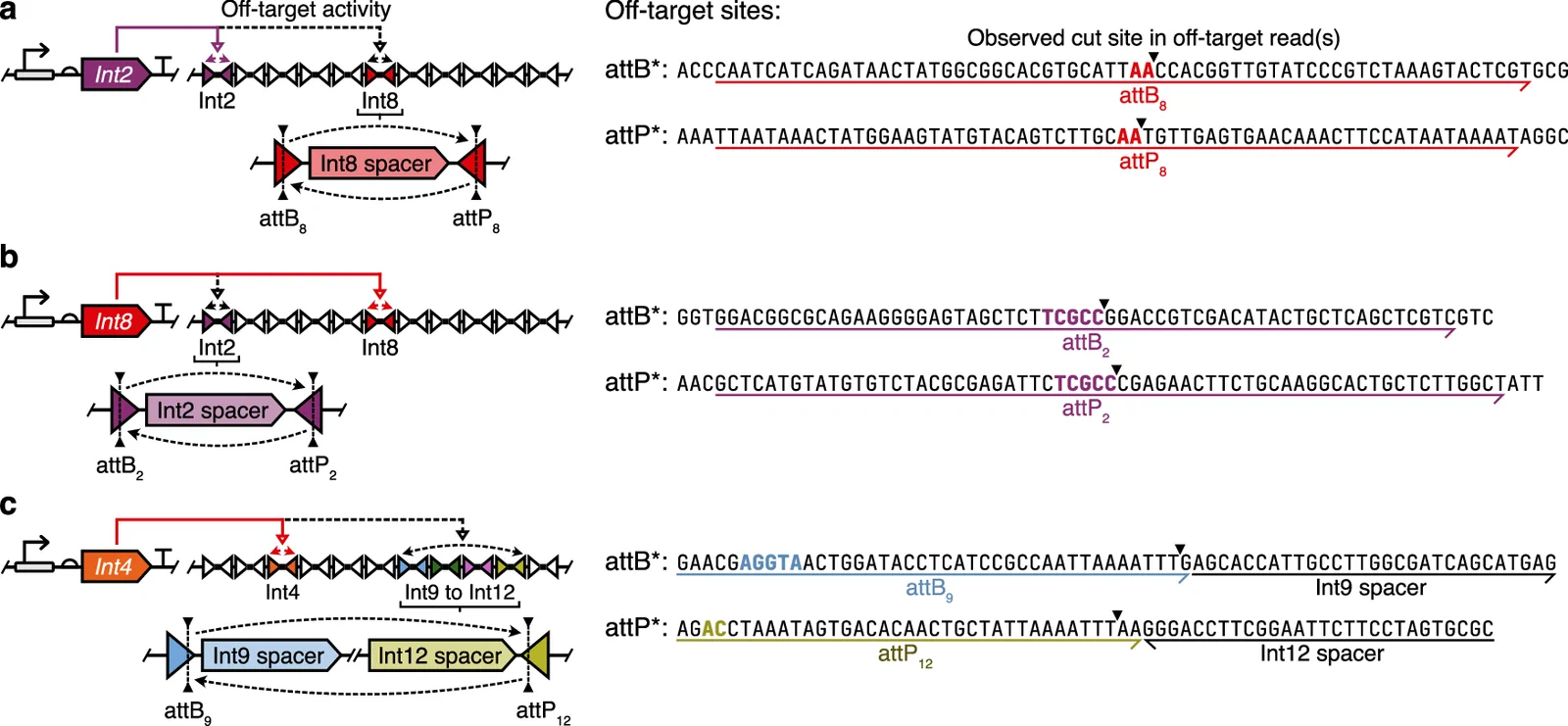
Nucleotide resolution insights into off-target recombinase activity. Analysis of reads where both the cognate region for an expressed integrase is flipped, in addition to a non-cognate site. Genetic architecture of the changes observed shown on the left and inferred attB and attP sequences for the off-target activity shown on the right. Cut site found from the sequencing data shown by a small black triangle. Annotations of known genetic sequences and their orientation from the pMemoryArray plasmid shown below each sequence. **a** Off-target activity for Int2 expression. **b** Off-target activity for Int8 expression. **c** Off-target activity for Int4 expression.

A further benefit of long-read sequencing for characterization is its ability to provide nucleotide-resolution insight. To demonstrate this ability, we collected reads in which both an expected (cognate) site and an additional off-target site were flipped. These reads were grouped according to the integrase expressed in each sample, and comparisons made to infer the precise points at which DNA had been cut at the off-target sites. This allowed us to infer sequences for which each integrase had an affinity, even when these were not its own cognate attB/attP sites (Fig. 3).

For samples where Int2 and Int8 were expressed, a single read from each sample contained both the Int2 and Int8 spacer sequences flipped (Fig. 3a, b). Analysis of the off-target activity in both cases showed that the cut site matched the expected cut site for that sequence and its cognate integrase (i.e., for Int2 expression, the Int8 attB and attP sites were cut precisely where Int8 would be expected to cut). As only a single read out of more than 100,000 showed this pattern in each case, this activity is very rare. More interestingly, for samples where Int4 was expressed, we observed the flipping of a region whose inferred attB and attP sites did not correspond to any known site for any of the integrases considered in this study (Fig. 3c). We classified three such reads across two biological replicates, suggesting that this activity was more common than other off-target activity observed and is unlikely to result from sequencing artifacts such as barcode misclassification. The flipped region was 592 bp long, beginning 1 bp before the end of the Int9 attB site and 2 bp from the end of the Int12 attP site.

Finally, we used our ability to pinpoint the bases where recombination occurs to also extract consensus cut sites for both the attP and attB sequences across all the integrases studied. As expected, we found that these matched cut sites previously documented in the literature (Supplementary Table 4)^21^.

### Observing multi-state dynamics of recombinase-based circuits

**Fig. 4:**
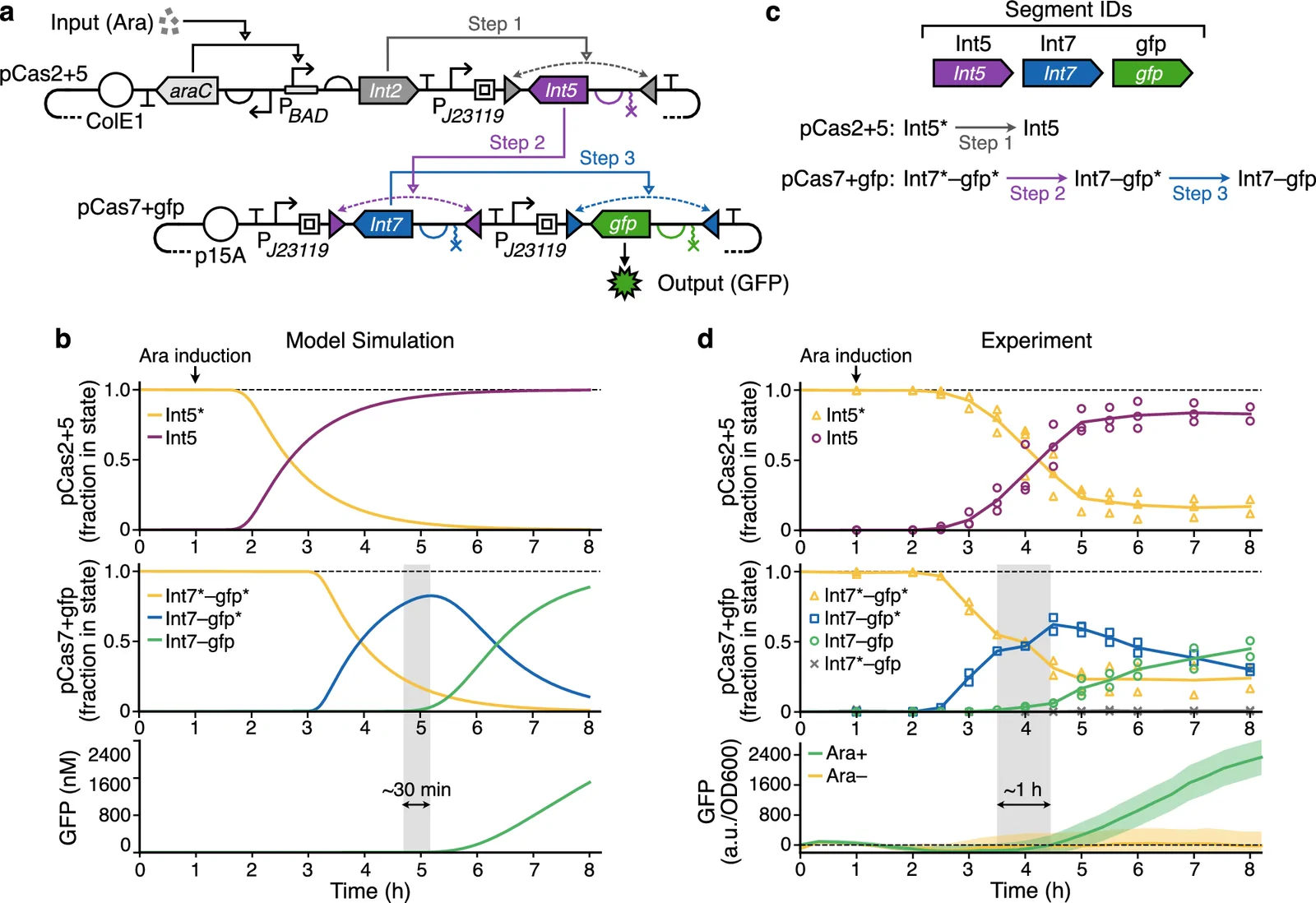
Monitoring of a multi-step recombinase-based cascade. **a** Schematic of the recombinase-based genetic cascade. The circuit spans two plasmids (pCas2+5 and pCas7+gfp) and requires the flipping of coding sequences of two recombinase enzymes (Int5 and Int7) and an output green fluorescent protein (GFP). The cascade is triggered by the presence of arabinose (Ara). **b** Numerical simulations of the mathematical model describing the dynamics of the recombinase-based cascade after induction at 1 hour. The top two plots show the fraction of reads for the pCas2+5 and pCas7+gfp plasmids in the different possible states over time. The bottom plot shows GFP output over time. Gray shaded period denotes the delay in the flipping of the *gfp* coding sequence in the pCas7+gfp plasmid and when a subsequent change in GFP expression is seen (~30 min). **c** Segments used for the analysis of circuit state. Transition steps between states for each of the plasmids are shown. **d** The top two plots show the fraction of reads for the pCas2+5 and pCas7+gfp plasmids in the different possible states over time. Solid lines show the average fraction and unfilled triangles, circles, squares, and crosses show the individual measurements taken for all three of the biological replicates. The bottom plot shows GFP output normalized to OD600 over time when the cascade is induced with arabinose (Ara+, green solid line) or not (Ara-, yellow solid). The standard deviation of the three biological replicates is shown by the colored shaded regions. Gray shaded period denotes the delay in the flipping of the *gfp* coding sequence in the pCas7+gfp plasmid measured by sequencing, and the subsequent change in fluorescence after the time taken to express the protein and for it to mature (~1 h). Source data for this figure is available in the Source Data file. Source code for simulations presented in this figure is available at GitHub.

Another major advantage of using long-read sequencing to monitor recombinase-based circuits is that more complex multi-state changes can be directly quantified in their entirety. To demonstrate this capability, we made use of a multi-component genetic cascade in which three recombinases trigger the sequential expression of one another through the structural rearrangement (flipping) of subsequent recombinase protein-coding sequences (Fig. 4a)^21^. A further complication of this system is that not all components are present on a single plasmid, but are instead split across two, offering the chance to assess our methodology when targeting multiple loci within a single genetic system.

To explore how this circuit might behave, we used the characterization data previously collected for the recombinases in isolation (Fig. 2) to develop a mathematical model of the circuit’s dynamics (*Methods*; Supplementary Data 3). The model was based on ordinary differential equations (ODEs), with variables capturing the fraction of flipped recombinase and GFP coding regions across the population, as well as the concentrations of the expressed recombinase and GFP proteins. The variables describing the flipped state of the recombinase and GFP coding regions corresponded directly to measurements that could be made using our sequencing approach. Where possible, we used parameters measured in our own experiments (i.e., from Fig. 2b), and biologically feasible values otherwise.

Numerical simulations of the model showed the expected sequential expression of the recombinases as their coding sequences were flipped, until the final output gene (*gfp*) was activated (Fig. 4b). The rate of flipping for each recombinase slowed over time because of the reduced concentration of unflipped substrate arising from the irreversibility of the process (e.g., see the Int5 and Int7-gfp states). We also observed a delay of ~30 min between flipping of the GFP coding region and notable increases in GFP protein expression, reflecting the time required to synthesize the protein. Note that our model did not account for maturation of GFP (~25 min for many GFP variants^34^), which is required for it to be monitored by fluorescence. Therefore, experiments would likely observe even longer delays between inversion of the GFP coding region and a measurable fluorescence output from the circuit.

To assess how well our model captured the dynamics of the circuit in vivo, we performed experiments in *E. coli* where the genetic cascade was triggered (at *t* = 0 h) and then the internal state of the system monitored through sampling of the culture over time. Samples were processed using our automated workflow with two separate PCRs to target the two relevant regions making up the full circuit (present on the pCas2+5 and pCas7+gfp plasmids, respectively). To quantify the circuit state, we used the two recombinase coding sequences that were initially flipped (Int5 and Int7) and the output green fluorescent protein (GFP) as segments to search for within each read (Fig. 4c). This allowed us to quantify the proportion of cells at each step in the cascade.

Analysis showed a clear propagation of inversions in the coding sequences of the recombinases and GFP over time that corresponded to the sequential expression of each recombinase in the cascade (Fig. 4d). The changes observed also qualitatively matched the response seen by our model. Similar to the characterization experiments of the recombinases in isolation, we saw increased rates of sequence inversion by Int5 compared to Int7. Interestingly, the dynamics of the cascade circuit are notably slower than those observed in the single-recombinase characterization experiments. This difference could stem from several sources. First, the initial characterization experiments expressed the recombinase from a higher-copy plasmid (ColE1 origin, ~20 copies per cell) than the target memory array (p15A origin, ~10 copies per cell), whereas many of the steps in the cascade encoded the recombinase expression cassette and its target DNA sites at identical copy numbers per cell (e.g., Int2 and Int7), effectively reducing the recombinase concentration per site and slowing the flipping rate. Second, switching each recombinase in sequence takes additional time, as expression of a newly activated recombinase must reach a sufficiently high concentration to efficiently trigger the next recombinase in the cascade. Finally, the context of the recombination sites also differs, with the distance between cognate sites being much greater than the 50 bp separating them in the memory array, potentially slowing the rate at which recombination proceeds. Furthermore, if the flipping of a segment is slow, unconverted plasmids may persist and continue to replicate as the experiment proceeds, resulting in a stable unflipped population of plasmids. Therefore, the dynamics we observe are also likely to depend on the relative rates of recombinase-mediated flipping and plasmid replication and dilution. This may account for the incomplete flipping of the *gfp* segment in the experiments (Fig. 4d), a result of flipping, replication and dilution occurring on similar timescales.

In addition to monitoring the state of the circuit DNA, the ability to take very small samples from a growing culture for monitoring also allowed us to perform these experiments in a plate reader and monitor GFP fluorescence throughout the experiment. Integrating this fluorescence data with the dynamics of the circuit state via sequencing, we were able to see a clear delay of ~1 h between changes in the orientation of the *gfp* gene and the subsequent fluorescence produced after gene expression is activated. This matched what would be expected based on results from our model and further highlights a key benefit that more direct sequencing-based measurements of circuit state have over more commonly used fluorescent reporter proteins. Specifically, recombination events are directly measured when they happen, rather than via delayed changes in fluorescence.

### Quantifying the spatial activation of recombinase-based switches in plants

A major benefit of recombinase-based circuits is their ability to function robustly across diverse organisms^10,35,36^. A particularly promising avenue of their use is tracking cellular decision-making during the development of more complex multicellular organisms and the creation of robust switches that can permanently turn on functionalities in specific cell types^37^. Currently, monitoring the state of these types of circuits is challenging. Fluorescence microscopy is often used^37^; however, this limits the number of states that can be simultaneously read and hampers accurate quantification of circuit state across large tissues, as cell boundaries are often difficult and time-consuming to delineate.

**Fig. 5:**
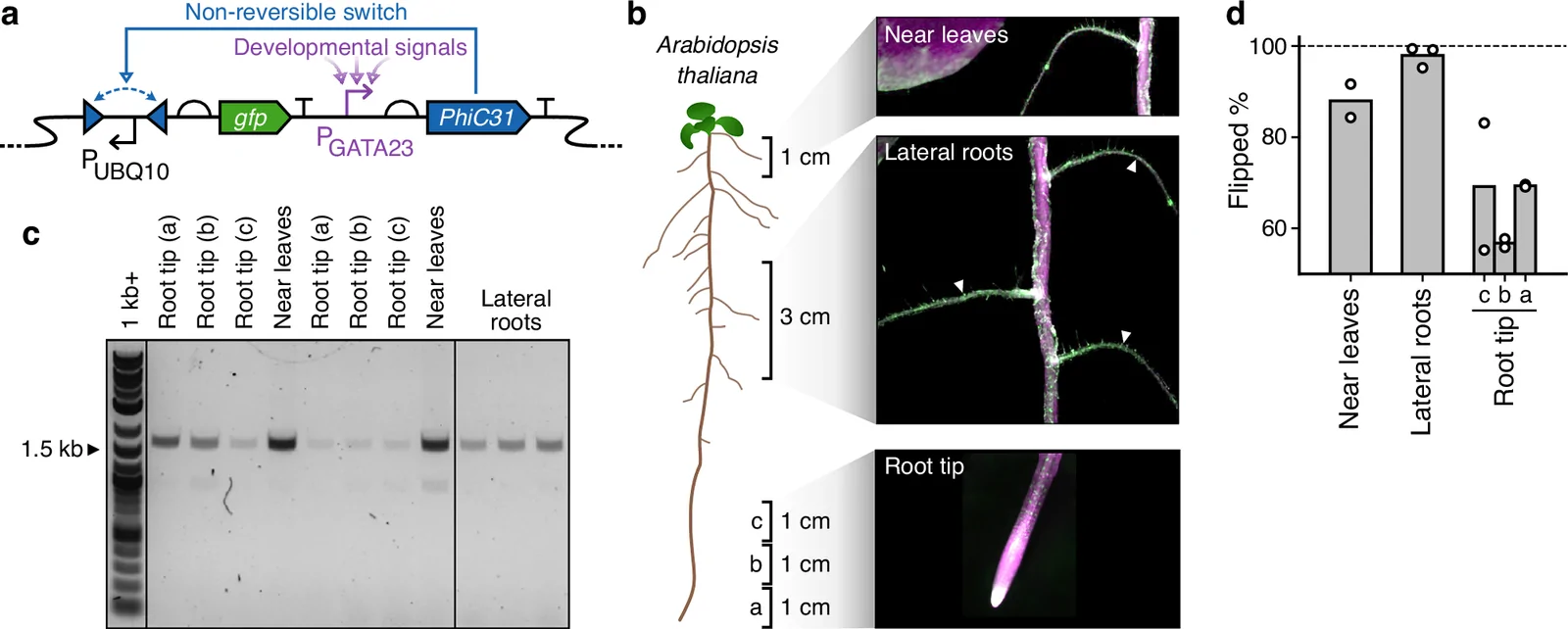
Monitoring spatial activation of a recombinase-based switch in plant roots. **a** Schematic of the recombinase-based genetic switch. Expression of the PhiC31 recombinase by the P_GATA23_ promoter (active in lateral roots) causes the non-reversible flipping of the P_UBQ10_ promoter, which is then able to drive green fluorescent protein (GFP) expression. **b** Sections of *Arabidopsis thaliana* roots that were dissected for analysis. White triangles denote the lateral roots that were separated from the primary root. **c** Tailed DNA from different sections of root after magnetic bead clean-up. Roots were cut into the sections illustrated in (**c**) and then placed directly in tailing PCR reactions. Magnetic bead clean-ups were carried out on the PCR products. The amplicon is expected to be ~1.5 kb in length. Gel shown for biological replicates (two for samples near leaves and from the root tip, and three for the lateral roots). **d** Activation of the genetic switch in different sections of the root. Values for biological replicates (two for samples near leaves and from the root tip, and three for the lateral roots) of the experiment are shown by white-filled circles. Source data for this figure is available in the Source Data file.

To demonstrate the ability of our workflow to overcome these challenges, we analyzed the spatial activation of a recombinase-based switch that had been integrated into the genome of *Arabidopsis thaliana* (Fig. 5a). The circuit was designed to strongly activate GFP expression in lateral roots. This was achieved by using the P_GATA23_ promoter (active in lateral root primordia^38^) to trigger the expression of the PhiC31 recombinase, which then permanently flips the orientation of a constitutive promoter to drive expression of GFP in primordia-derived cells (i.e., lateral roots).

To assess activation of the circuit throughout the root system, we dissected three key areas: (i) a 1 cm section of the primary root near the leaves, (ii) lateral roots in a 3 cm section in the middle of the root system, and (iii) the root tip into three separate 1 cm sections (Fig. 5b; *Methods*). A significant issue we faced when working with this dissected material was the very small amounts of DNA that could be recovered. The use of PCR in our workflow enabled us to target and amplify the specific regions of interest. However, optimization of the conditions and polymerases used in this reaction was required before amplicons were consistently recovered for the tailing PCR step (Fig. 5c; *Methods*).

As expected, analysis of the circuit showed near full activation (98%) in lateral roots (Fig. 5d). The lowest activation was seen in the root tip (56–69%) and slightly higher activation in the primary root near the leaves (88%). This qualitatively matched the clear expression of GFP throughout lateral roots when imaged by microscopy (Fig. 5b). We also found fairly consistent values across biological replicates of the experiment. The quantitative difference we observed in activity between the primary root near the leaves and the root tip is likely due to low basal expression from the P_GATA23_ promoter. Because cells near the leaves have lived for longer than those in the root tip, there is a higher chance of permanent activation in the cells near the leaves due to stochastic gene expression events (e.g., transcriptional bursts) over time.

## Discussion

Recombinase-based genetic circuits offer much promise for the robust reprogramming of diverse organisms^35^. However, for this to become a reality, many researchers have highlighted the need for efficient and comprehensive approaches for reading and interpreting the state of these circuits^27,39,40^. Here, we have addressed this need, showing how a semi-automated experimental and computational workflow can enable high-throughput monitoring of entire recombinase-based circuits with minimal requirements in terms of starting DNA material and no requirements to make changes to the circuits themselves. Furthermore, the ability of nanopore sequencing to be carried out in the field at the point of use^41^ opens up new avenues for monitoring these circuits no matter where they are deployed.

To date, over 4000 putative large-serine recombinases have been identified, but the vast majority of these remain uncharacterized^21,35,42^. A better understanding of recombinase diversity would significantly expand the toolkit of parts available for synthetic biologists and offer a more systematic understanding of their function across the tree of life. Our workflow is ideally suited to address this need. It allows time-course experiments to be sampled to recover reaction kinetics, and the use of large arrays of diverse recombinase recognition sites allows numerous off-target activities to be characterized simultaneously. Combining our sequencing approaches with high-throughput chip-based DNA synthesis would enable an even more comprehensive understanding of how these molecular machines work and generate ideal data sets to train AI models for future de novo design of these molecular machines.

A major advantage of our approach for classifying read configurations using short, user-defined ‘segments’ is that it removes the need to generate reference sequences for every possible circuit configuration. Mapping-based approaches, which rely on tools such as minimap2, require a full reference sequence in order to find the most probable alignment for each read. This is feasible for the genetic memory array considered here (Fig. 2), which has 2048 possible configurations. However, as circuit complexity grows, enumerating every reference sequence quickly becomes intractable due to memory constraints. A recombinase circuit with just 30 independently invertible regions already has over 1 billion possible configurations, and this number grows far more rapidly when overlapping inversions, excisions and insertions are possible. The problem becomes insurmountable for larger systems such as the engineered Sc2.0 genome^43,44^. When complete, this genome will contain approximately 3000 DNA segments flanked by loxPsym sites that enable large-scale chromosome rearrangements. Because chromosomes can expand during recombination procedures like SCRaMbLE^44^, the number of possible configurations is unbounded, and so reference-based approaches cannot be applied at all.

In this work, we overcome this issue of combinatorial explosion by instead analyzing the order and orientation of key segment sequences. This allows us to classify reads efficiently without any complete reference for the system. The resulting data also remain highly interpretable, making it possible to pinpoint the root cause of failures such as unexpected duplications and deletions arising from circuit instability^45,46^. In addition, because the segments used to characterize a construct’s state typically tile the entire circuit, it is straightforward to zoom in on the most relevant region of interest and extract its sequences for deeper analysis. This is precisely how we revealed the off-target attB/attP sites responsible for the unexpected flipping of a large region of the memory array (Int9–Int12) during Int4 expression (Fig. 3). Furthermore, many genetic parts and circuits, including recombinases, self-cleaving ribozymes, transcriptional terminators and introns, work by altering the architecture (i.e., the presence, order and orientation of sub-sequences) of DNA or RNA molecules. Our segment-based approach should therefore prove effective across a wide range of other challenging characterization tasks in synthetic biology.

While our methodology offers insights into recombinase part and circuit function, there are several remaining challenges. First, although automating the preparation of sequencing libraries substantially reduces hands-on time, operator error, and per-sample cost, the overall cost of the workflow remains non-trivial and greater sample multiplexing would be beneficial. It is useful to consider where these costs lie, because the two main levers for reducing them act on different components. As the per-base cost of sequencing has continued to fall, the reagents and consumables used in library preparation have come to represent a growing, and in many workflows dominant, fraction of the total expense^47^. In our workflow, however, the single largest contribution still comes from the flow cell (*Methods*). Therefore, the effective cost per sample is strongly influenced by how many barcoded samples can be pooled on each flow cell. Increasing the level of multiplexing offers the most direct route to lowering this component of the cost, as seen in the roughly proportional reduction in sample cost when moving from 24 to 192 samples per MinION flow cell (*Methods*). However, this gain is bounded because each sample must still have sufficient reads to confidently determine its circuit state (typically more than 25,000), and we found that running more than 48 samples per flow cell could leave some samples under-sequenced when the quality of input DNA was variable. The practical ceiling on multiplexing is thus set by the interplay between total flow cell output and the read depth required per sample, which itself scales with circuit complexity. Matching sequencing capacity to experimental scale and using higher-output platforms like PromethION for large batches or Flongles for smaller numbers of samples provides additional flexibility that can be exploited during experimental design. We have also observed continued increases in read quality and total number of reads per flow cell as the nanopore sequencing technology has matured, further lowering overall costs.

Reducing the volume of each reaction offers a complementary route that targets the reagent and consumable portion of the cost. The positive-displacement, tip-based liquid handling used in our workflow (and in most automated protocols) is effective but carries appreciable reagent dead volumes, consumes large amounts of relatively expensive consumables (i.e., plastic tips), and does not miniaturize below the microliter scale^47^. Acoustic-based liquid handling overcomes these limitations by using focused ultrasound to transfer nanoliter droplets without physical contact. This allows for reaction volumes to be reduced by one to two orders of magnitude while retaining data quality equivalent to full-volume reactions. This approach has been used to miniaturize and automate next-generation sequencing library preparation, cutting reagent costs by over 80% and preparation time by around 90% relative to manual workflows^47^, as well as to prepare plasmid DNA libraries for sequencing-based quality control^48^ and to downscale the PCR and one-pot DNA assembly reactions (e.g., Golden Gate and Gibson) that underpin many synthetic biology experiments, with reagent costs reduced 20- to 100-fold^49^. Adapting the tailing PCR, barcoding PCR, and concentration-normalization steps of our workflow to a nanoliter-scale acoustic platform could therefore significantly lower reagent consumption. A further benefit would be a reduction in single-use plastic, which is a growing issue for laboratories aiming to adopt sustainable practices^50^.

A second challenge is that our reliance on PCR to both target and amplify the relevant regions of a recombinase circuit could, in principle, distort the measured relative abundances of different circuit states. Quantitative PCR from a mixed pool of templates is known to be susceptible to preferential amplification, whereby the composition of the final amplicon pool no longer faithfully reflects the input^51,52^. It is therefore worth considering the extent to which such biases might affect our measurements and where they are most likely to arise.

One of the dominant sources of bias in multi-template PCR stems from differences in primer-template binding kinetics. When the templates in a reaction differ across their primer-binding sites (e.g., through sequence divergence or the use of degenerate primers) they anneal to the primers with different efficiencies and binding energies, with more efficiently primed templates becoming progressively over-represented each cycle^52,53^. Even a single primer-template mismatch can substantially skew the relative amplification of otherwise similar templates^54,55^. Our workflow is designed to avoid this class of bias entirely. Because recombination acts on the interior of the circuit while leaving the flanking regions untouched, the same pair of tailing primers bind the same invariant sequences irrespective of circuit state, so every state is primed with identical kinetics and no state is favored at the priming step. The subsequent barcoding PCR likewise primes from the common tails shared by all amplicons, and because a given barcode is applied per sample rather than per state, it cannot distort the relative proportions of states measured within that sample.

The bias that our design does not eliminate arises later, during elongation and amplification, and is driven by properties of the amplicon itself (e.g., base composition and length). Templates with extreme GC content are amplified less efficiently, because GC-rich duplexes are harder to denature and can form secondary structures that affect polymerase progression, while very AT-rich regions are similarly prone to under-representation. Due to these effects, PCR during library preparation has been identified as the principal source of this base-composition bias in sequencing data^56,57^. Length introduces a second effect: shorter amplicons are amplified more efficiently than longer ones and, in a mixed reaction, out-compete longer templates, causing shorter states to become over-represented. For example, size-dependent template competition has been shown to strongly suppress the recovery of longer fragments in length-polymorphic genotyping and metabarcoding^58–60^. Importantly, these effects are negligible for the circuits characterized here. All of the recombination events we measure, i.e., the flipping of segments in the memory array (Fig. 2), the inversion of recombinase and reporter coding sequences in the cascade (Fig. 4), and the flipping of the promoter in the plant switch (Fig. 5), are inversions that leave the length and overall base composition of the amplified region unchanged between states. The two orientations of an inverted segment should therefore be amplified with near-identical efficiency, and the strong agreement we observe between expected and measured flipped fractions across the full potential range is consistent with this expectation. However, composition- and length-dependent bias becomes a genuine concern for circuits in which recombination more substantially alters the amplicon itself. For example, circuits employing excisions or insertions that shorten or lengthen the region between the tailing primers, where a shorter state could become over-represented relative to a longer one.

Where such length or composition differences are unavoidable, several strategies can reduce or correct the residual bias. Because bias accrues disproportionately in the later, more competitive cycles of the reaction, minimizing the cycle number, increasing reaction volumes, and pooling replicate reactions all help to keep the product distribution closer to the template distribution^51,52^. Incorporating unique molecular identifiers into the tailing primers would allow individual template molecules to be counted after amplification, correcting for uneven copying directly at the analysis stage^61^. More fundamentally, the amplification step can be reduced or removed altogether by enriching the target DNA through hybridization capture with biotinylated probes^62^ or Cas9-directed cleavage^63^, or by attaching barcoded sequencing adapters through direct ligation or transposase-based tagmentation (e.g., using rapid barcoding kits for sequencing library preparation) rather than a second PCR^64^. These amplification-free routes are particularly attractive for circuits whose states differ substantially in length, and represent a natural elaboration of our workflow for such use cases.

In summary, recombinase-based genetic circuitry offers bioengineers the rare ability to create reliable genetic logic and memory due to its direct encoding of discrete states within the structural organization of a circuit’s own DNA. Scaling our ability to build and characterize such systems will be critical to their use in applications spanning agriculture to health^1^. This work provides the integrated experimental and computational tools needed to take steps towards this goal, helping broaden the metrology available for understanding large and complex genetic circuitry^15,65–67^, and ultimately supporting improved robustness in the engineered biology we create.

## Methods

### Strains, media, and chemicals

Cloning and characterization experiments in bacteria were performed using *E. coli* strain DH10-beta (Δ(ara-leu) 7697 araD139 fhuA ΔlacX74 galK16 galE15 e14- *ϕ*80dlacZΔM15 recA1 relA1 endA1 nupG rpsL (StrR) rph spoT1 Δ(mrr-hsdRMS-mcrBC)) (C3019, New England Biolabs). *E. coli* were grown in NEB 10-beta stable outgrowth medium (B9035S, New England Biolabs) for transformation, and LB broth (L3522, Sigma-Aldrich) for general propagation and characterization. Antibiotic selection was performed using 50 μg/ml Chloramphenicol (C0378-25G, Sigma-Aldrich) and 50 μg/mL kanamycin (K1637, Sigma-Aldrich). Induction of circuit components for the recombinase characterization and cascade experiments was performed using 10 mM of L-Arabinose (1B1473-25G, VWR), and for samples where no induction was present, cells were grown in LB media supplemented with 0.5% D-glucose (G7528, Sigma-Aldrich) to ensure strong catabolite repression of the P_BAD_ promoter.

### Transgenic plants

Transgenic plants were generated by *Agrobacterium tumefaciens*-mediated floral dip of *Arabidopsis thaliana* Columbia ecotype^68^. Transgenic seeds contain a constitutively expressed mCherry marker *proUBQ10*::*mCherry* that was used to isolate transgenic seeds via fluorescence microscopy. A single insertion homozygous transgenic line was isolated and used for switch analysis *in planta*.

### Plant growth conditions

Transgenic *Arabidopsis thaliana* plants were grown in Percival-Scientific growth chambers (CU41L4) at 22 ^∘^C in 16/6-h light/dark cycles with 60% humidity. The light was provided by LED arrays at a total intensity of ~100 microEinsteins (μE). Plants for imaging and switch analysis were plated as seeds onto sterile 0.7% gelzan (G19010, Sigma-Aldrich) media containing 1X Murashige and Skoog nutrients (MSP01-50LT, Caisson) and 1% sucrose (S0389, Sigma-Aldrich) at pH 5.7. Prior to plating, seeds were surface sterilized prior to plating with 95% ethanol and 20% bleach. Plates were sealed with micropore tape (1530-0, 3M) for gas exchange and placed vertically into the growth chamber.

### Assembly of pSpacerX plasmids

The pSpacerX plasmids were assembled in a 2-step cloning process. First, a 4-part Golden Gate reaction was used to create a template plasmid with a 1 kb spacer flanked by Int2-attP and Int2-attB sites. This reaction was composed of a destination backbone (p15A-CamR), two short fragments with the Int2-attP and Int2-attB sequences designed to flank the spacer, and a 1 kb inert spacer sequence derived from the LacI protein coding sequence. This plasmid was then used as a template for full-plasmid PCR and blunt-end ligation using a series of oligos that would truncate the LacI sequence, resulting in spacers of the desired lengths. For the Golden-Gate reaction, PCR fragments were generated from pMemoryArray for the plasmid backbone, as well as the Int2-attP and Int2-attB recognition sequences, and the 1 kb LacI spacer was amplified from the *E. coli* genome. pMemoryArray was a gift from Christopher Voigt (Addgene plasmid #60585). The NEBridge Golden Gate Assembly Kit (BsaI-HF v2) (E1601, New England Biolabs) was used for the Golden-Gate assembly reaction using standard conditions, except reaction volumes were scaled to a quarter of the normal volume. For the subsequent blunt-end ligation cloning, a KLD Enzyme Mix (M0554, New England Biolabs) was used following the standard manufacturer protocol. For all assembly reaction transformations, 12.5 μL of chemically competent DH10-beta cells (C3019, New England Biolabs) were transformed with 2 μL of the assembly reactions. All plasmid sequences were verified by Sanger sequencing (Eurofins Genomics).

### Recombinase characterization experiments

For experiments performing a detailed characterization of a set of recombinases (Fig. 2), *E. coli* DH10-beta competent cells were transformed with both a plasmid expressing a recombinase (pIntegrase_2–8; #60574–60579, Addgene) and an 11-bit Memory Array (pMemoryArray; #60585, Addgene), which were gifts from Christopher Voigt. For the experiments with varying segment lengths (pSpacerX plasmids), *E. coli* DH10-beta competent cells were transformed with both the pIntegrase2 plasmid and a specific pSpacerX plasmid with the appropriate segment length. Single colonies of transformed cells were then inoculated in 5 mL of LB media supplemented with kanamycin, chloramphenicol, and 0.5% glucose to suppress expression of the P_BAD_ promoter. Cultures were grown overnight for 15 h in a shaker incubator (SI500, Stuart) at 37 ^∘^C and 250 rpm. After the overnight growth, measurements of OD600 were taken using a WPA Biowave II spectrophotometer to assess cell growth. For induction, pre-cultures were diluted by the appropriate factor to an OD600 of 0.3 in 3 mL of media supplemented with kanamycin and chloramphenicol, and 0.5% glucose to maintain minimal expression for uninduced samples. These experiments were conducted in 24-deep well plates (V6831, Promega UK) at 37 ^∘^C and 1250 rpm in a shaker incubator (SI505, Stuart). Recombinase expression was induced using 10 mM L-arabinose, after 1 h of initial incubation. For the initial characterization experiments, 25 μL samples were collected and flash-frozen at 0, 40, 80, 120, 150, 180, 210, 240, and 360 min after induction. For the second characterization experiment, 25 μL samples were collected and flash-frozen at 20, 30, 40, 50, 60, 70, 80, 90, 100, and 160 min after induction. For the experiments with varying segment lengths, the same protocol was followed; however, 25 μL samples were collected and flash-frozen at 70, 80, 90, 100, 110, 120, 130, 140, 150, and 210 min after induction with 10 mM L-arabinose. To assess the accuracy of the workflow using mixtures of unflipped and flipped memory arrays, we grew separate 5 mL cultures in LB broth. One culture harbored the original, unflipped pMemoryArray plasmid; the other harbored the same plasmid after the Int2, Int5, Int7 and Int8 regions had been fully flipped by exposure to the relevant integrases (hereafter the ‘flipped plasmid’). Cultures were grown to an OD600 of 0.8, and any culture exceeding this value was diluted back to 0.8. We then prepared 1 mL mixed cultures containing 0%, 2.5%, 5%, 50% and 100% cells harboring the flipped plasmid. These were vortexed thoroughly to ensure even mixing, and 25 μL samples were collected and flash-frozen for processing. All samples across experiments were immediately stored at −80 ^∘^C before processing using the automated workflow (Supplementary Note 1) using tailing primers specific for the plasmids present (Supplementary Table 5).

### Mathematical model of the recombinase-based cascade

We developed an ODE model to capture the dynamics of the recombinase (Int2, Int5 and Int7) and GFP protein concentrations, together with the state of the recombination sites (i.e., their flipped fraction across the population), within the two-plasmid genetic circuit shown in Fig. 4a. All concentrations are given in nM, time is measured in hours (h), and all concentrations and flipped fractions are set to zero at *t* = 0.

To begin, the concentration *c*_2_ of the input recombinase Int2 was described by

1 $$\frac{d{c}_{2}}{dt}\,=\,{\alpha }_{2}\,u(t)-{\gamma }_{2}{c}_{2},$$

where *α*_2_ is the production rate of Int2 (nM/h), *γ*_2_ is its combined degradation and dilution rate (h^−1^), and *u*(*t*) is an input function that governs when Int2 expression is induced (i.e., upon addition of arabinose), given by

2 $$u(t)\,=\,\left\{\begin{array}{ll}\,0\quad &\,{\mbox{if}}\,t < 1,\\ \,1\quad &\,{\mbox{otherwise}}\,.\end{array}\right.$$

Induction therefore begins at *t* = 1 h. The concentrations of Int5, Int7 and GFP are tracked by the variables *c*_5_, *c*_7_ and *c*_*G*_, respectively, with dynamics given by

3 $$\frac{d{c}_{5}}{dt}\,=\,{\alpha }_{5}{f}_{5}-{\gamma }_{5}{c}_{5},$$

4 $$\frac{d{c}_{7}}{dt}\,=\,{\alpha }_{7}{f}_{7}-{\gamma }_{7}{c}_{7},$$

5 $$\frac{d{c}_{G}}{dt}\,=\,{\alpha }_{G}{f}_{G}-{\gamma }_{G}{c}_{G}.$$

Here, *α*_5_, *α*_7_ and *α*_*G*_ are the production rates of Int5, Int7 and GFP when their coding regions are in the flipped orientation; *γ*_5_, *γ*_7_ and *γ*_*G*_ are the corresponding combined degradation and dilution rates; and *f*_5_, *f*_7_ and *f*_*G*_ are the fractions of the respective coding regions across the whole population that are in the flipped orientation. Because the recombinase and GFP proteins are highly stable, we assume that dilution through cell replication dominates their loss and set all *γ* parameters to 0.5 h^−1^, reflecting the approximately 30 min doubling time of the *E. coli* cells used. We set the production rates *α* based on the recombination rates observed in Fig. 2, accounting for the fact that both Int2 and Int5 are encoded on a higher-copy ColE1 plasmid (~20 copies per cell), whereas Int7 and GFP are encoded on a lower-copy p15A plasmid (~10 copies per cell). We further assume that the output GFP is produced at a much higher rate than all the recombinases, due to the strong ribosome-binding site and the stabilizing RiboJ self-cleaving ribozyme^69^ present in that transcriptional unit. Specifically, we use *α*_2_ = *α*_5_ = 100 nM/h, *α*_7_ = 50 nM/h, *α*_*G*_ = 1000 nM/h, and *γ*_2_ = *γ*_5_ = *γ*_7_ = *γ*_*G*_ = 0.5 h^−1^.

Recombination (i.e., flipping) of a region of DNA requires assembly of a synaptic complex, with an integrase dimer bound at each site and the two dimers associating into a tetramer that bridges the sites. We assume that this binding and synapsis equilibrate rapidly relative to both catalysis and recombinase turnover, causing a separation of timescales. Under a quasi-steady-state assumption, the probability that an unflipped copy occupies a catalytically competent synaptic state can be written as a function of the integrase concentration, *R*, and the cooperativity of recombinase assembly, *n*, giving the Hill equation

6 $${p}_{{{{\rm{syn}}}}}(R)\,=\,\frac{{R}^{n}}{{K}^{n}+{R}^{n}},$$

where *K* is the recombinase concentration giving half-maximal synapse formation. Because both sites lie on the same molecule, inversion is intramolecular. Therefore, once the synaptic complex has formed, flipping proceeds as a first-order event per copy, with no bimolecular DNA–DNA term. Each unflipped copy is therefore converted to a flipped state at a rate

7 $$\lambda (R)\,=\,{k}_{{{{\rm{cat}}}}}\,{p}_{{{{\rm{syn}}}}}(R),$$

where *k*_cat_ is the maximal per-copy recombination rate at saturating recombinase concentration.

Considering a mean-field balance for the flipped fraction across the population, the unflipped pool (1 − *f*) is converted at rate *λ*(*R*) and, because the reaction is unidirectional, there is no reverse flux, giving

8 $$\frac{df}{dt}\,=\,\lambda (R)\,(1-f).$$

In the cascade, each recombinase catalyzes flipping of the coding region for the next stage: input Int2 (*c*_2_) flips the Int5 region (*f*_5_), Int5 (*c*_5_) flips the Int7 region (*f*_7_), and Int7 (*c*_7_) flips the GFP region (*f*_*G*_). Applying the balance above to each stage therefore gives

9 $$\frac{d{f}_{5}}{dt}\,=\,{k}_{{{{\rm{cat2}}}}}\,\frac{{c}_{2}^{\,{n}_{2}}}{{K}_{2}^{\,{n}_{2}}+{c}_{2}^{\,{n}_{2}}}\,(1-{f}_{5}),$$

10 $$\frac{d{f}_{7}}{dt}\,=\,{k}_{{{{\rm{cat5}}}}}\,\frac{{c}_{5}^{\,{n}_{5}}}{{K}_{5}^{\,{n}_{5}}+{c}_{5}^{\,{n}_{5}}}\,(1-{f}_{7}),$$

11 $$\frac{d{f}_{G}}{dt}\,=\,{k}_{{{{\rm{cat7}}}}}\,\frac{{c}_{7}^{\,{n}_{7}}}{{K}_{7}^{\,{n}_{7}}+{c}_{7}^{\,{n}_{7}}}\,(1-{f}_{G}).$$

The Hill coefficients were chosen to match the values measured when characterizing the recombinases in isolation (Fig. 2b), giving *n*_2_ = 10, *n*_5_ = 12 and *n*_7_ = 7. A maximal per-copy recombination rate of *k*_cat_ = 1 h^−1^ was used for all three recombinases (*k*_cat2_ = *k*_cat5_ = *k*_cat7_ = 1 h^−1^), estimated from in vitro measurements of comparable magnitude for other recombinases^70^. Finally, guided by literature values of 20–100 nM for the concentration required to reach half-maximal synapse formation in other recombinases^71^, the *K* value for Int2 was set to *K*_2_ = 75 nM, with *K*_5_ = 45 nM and *K*_7_ = 50 nM chosen to reflect the relative differences in *K* observed in our own characterization of the recombinases in isolation (Fig. 2b). Python code implementing the full model with an explicit Runge–Kutta method is provided as Supplementary Data 3.

### Experiments with a recombinase-based cascade

For experiments studying the recombinase-based cascade (Fig. 4), *E. coli* DH10-beta competent cells were transformed with two plasmids encoding a three-integrase cascade, which were gifts from Christopher Voigt (pCas2+5 and pCas7+gfp; #60594–60595, Addgene). Single colonies of transformed cells were then inoculated in 5 mL of LB media supplemented with kanamycin and chloramphenicol and 0.5% glucose to suppress expression of the P_BAD_ promoter. Cultures were grown overnight for 15 h in a shaker incubator (SI500, Stuart) at 37 ^∘^C and 250 rpm. After the overnight growth, measurements of optical density at 600 nm (OD600) were taken using a WPA Biowave II spectrophotometer to assess cell growth. For induction, pre-cultures were diluted by the appropriate factor to an OD600 of 0.3 in 3 mL of media supplemented with kanamycin and chloramphenicol, and 0.5% glucose to maintain minimal expression for uninduced samples. These experiments were conducted in 24-deep well plates (V6831, Promega UK) at 37 ^∘^C and 1250 rpm in a shaker incubator (SI505, Stuart). Recombinase expression was induced using 10 mM L-arabinose after 1 hour of initial incubation and 25 μL samples were collected and flash-frozen at 60, 120, 150, 180, 210, 240, 270, 300, 330, 360, 420 and 480 min. All samples were immediately stored at −80 ^∘^C before processing using the automated workflow (Supplementary Note 1) using tailing primers specific for the plasmids present (Supplementary Table 5).

### Experiments monitoring the spatial activation of recombinase switches in plants

For experiments monitoring the spatial activation of genetic switches in plant roots (Fig. 5), we made use of *A. thaliana* plants that contained a stably integrated recombinase switch for monitoring lateral root development (Fig. 5a). This strain had been generated via floral dip, with the first generation of seeds (T1) having been collected from selective media supplemented with antibiotic. These seeds were used to generate a second generation of transformant seeds (T2) that acted as the basis of our experiment. T2 *A. thaliana* seeds were first sterilized using ethanol to avoid bacterial and fungal contamination. Around 50 seeds were poured into a 1.5 mL microfuge tube and 1 mL of 70% ethanol was pipetted into the tube. The microfuge tube containing the seeds was incubated for 20 min on a tube rotator at room temperature. The ethanol was then removed by pipetting and particular care was taken to ensure that no residues were left. Following this, 1 mL of 95% ethanol was used to carry out a final wash of the seeds. After adding the ethanol, the seeds were incubated for 5 min on a tube rotator at room temperature. The tube containing the sterilized seeds was then transferred to a bio-safety cabinet. Ethanol was then removed by pipetting and 1 mL ultrapure water was added to the seeds to facilitate plating. Plating was done immediately after adding the water to avoid the water imbibing the seeds or causing them to stick together. Seeds were sown onto sterile plates containing 0.7% Gelzan (G19010, Sigma), 1X Murashige and Skoog nutrients (MSP01-50LT, Caisson), and 1% sucrose (S0389, Sigma) at pH 5.7. The pH was adjusted using KOH. Seeds were dispensed 1.5 cm apart to facilitate visualization and cutting of the seedling roots. Plates were sealed with breathable micropore tape (1530-0, 3M) to facilitate gas exchange and incubated in the dark for 72 h at 4 ^∘^C, kept vertical at all times. Plates were subsequently transferred to a culture growth chamber and grown for 10 days at 22 ^∘^C with cycles of 16 h of light and 8 h of dark, with 60% humidity levels. Light was provided by LED arrays at a total intensity of ~100 μE.

For microscopy, 10 days after sowing, the plants were imaged using a Leica Thunder Model Organism widefield fluorescence microscope. Excitation of fluorophores was performed using 450/40 nm excitation and 525/50 nm emission filters and 546/11 nm excitation and 605/70 nm emission filters for GFP and mCherry, respectively.

After testing several methods based on chemical and physical DNA extraction, we found that direct use of dissected material in a 50 μL tailing PCR reaction using the KAPA Taq HotStart PCR Kit (KK1512, Kapa Biosystems) performed well. This approach was used for all experiments with plant roots.

### Automated experimental workflow

Automated protocols were developed for the Opentrons OT-2 (Opentrons Inc.) liquid handling robot to carry out cell lysis and multiplexed sequencing library preparation (Supplementary Note 1; Supplementary Data 1). These protocols allow for the processing of 96 samples in parallel and generate two separately barcoded sequencing libraries. We found that running more than 48 samples per flow cell was possible, but that variation in run-to-run throughput (typically caused by the quality of the input DNA) could result in some samples having insufficient reads to accurately assess the circuit state. The total cost of running 96 samples through the automated experimental workflow was approximately £5.89 per sample (excluding the costs of the sequencing kits and flow cells) with a total runtime on the OT-2 of 13 h and 39 min (excluding robot setup and calibration time). Flow cell and sequencing library preparation costs per sample scale based on the level of multiplexing that is performed. For 24, 48, 96 and 192 samples per MinION flow cell, the cost per sample is approximately £32.84, £16.42, £8.21 and £4.10, respectively, with these costs assuming full UK prices. These costs are reduced significantly (>30%) if bulk purchasing of flow cells is performed as these are where most of the sequencing costs lie. Detailed information about each automated protocol can be found in Supplementary Note 1 and the optimization of these protocols is described in Supplementary Note 2. All OT-2 protocols were written using Python version 3.9.6 and the Opentrons API version 2.11. For lower-throughput requirements, we also provide descriptions in Supplementary Note 3 of manual protocols to carry out the experimental workflow.

In all of our experiments, heat lysis of the cells was conducted using the Temperature Module with PCR Aluminium Block (GEN2, Opentrons) and all PCR reactions were completed using the on-deck Thermocycler (GEN1, Opentrons). Both DNA purification steps were completed using the Magnetic Module (GEN2, Opentrons) with KAPA pure magnetic beads (KK8002, KAPA Biosystems) at a 0.4:1 bead-to-sample ratio. Opentrons GEN2 electronic pipettes were used during the pipeline (Single channels: P20, P300, P1000. Multi-channels: P20, P300). Supplementary Table 1 summarizes the steps involved in the automated pipeline, with protocol script numbers, modules, and pipettes that were used.

All DNA quantification for the automated workflow was performed using the Quantifluor ONE dsDNA system (E4870, Promega) in two 96-well plates. Standards were pre-prepared using the OT-2 robot according to the standard curve recommended by the manufacturer. Specifically, 1 μL of each sample was added to 200 μL of Quantifluor Dye. This was mixed and incubated for 5 min at room temperature and protected from light before being read using a SpectraMax plate reader (ID5, Molecular Devices) with 485 nm excitation and 535 nm emission filters. Analysis was performed using the Quantifluor Dye Systems Data Analysis Excel Workbook provided by Promega. Sequence-specific tailing primers were synthesized by IDT (Supplementary Table 5). Tailed samples were barcoded using a PCR Barcoding Expansion 1-96 (EXP-PBC096, Oxford Nanopore Technologies) to allow the pooling and running together of 48 sample sequencing libraries. Final nanopore sequencing libraries were generated using the standard ligation sequencing kit (SQK-LSK109, Oxford Nanopore Technologies).

### Nanopore sequencing

DNA sequencing libraries were sequenced using R9.4.1 flow cells (FLO-MIN106D, Oxford Nanopore Technologies) on a MinION Mk1B device (Oxford Nanopore Technologies) controlled using MinKNOW version 21.11.7. The resulting FAST5 sequencing data were basecalled and demultiplexed with guppy version 6.5.7 using the ‘dna_r9.4.1_450bps_hac.cfg’ high-accuracy model and ‘EXP-PBC096’ barcode kit to generate sample-specific FASTQ files that could then be used as input for downstream analyses.

### Segment analysis

We developed a tool called ‘segment’ to classify the configuration of the long nanopore reads from our experiments (Supplementary Data 2). The tool is written in the Rust (edition 2021) programming language and depends on the ‘bio’ version 1.0 and ‘clap’ version 4.5.9 crates for efficient semi-global alignment of short segment sequences and for command-line argument handling, respectively. The tool is run through a command-line interface (CLI) and takes three inputs: a set of reference sequences (the segments to search for) in multi-FASTA format; the minimum and maximum length of a valid read (e.g., to filter out short contaminants and long concatemer reads); and the reads to be processed in FASTQ format. Each read is then processed to generate its segment classification. Processing begins by searching the read for the special ‘start’ and ‘end’ segment sequences, which must always be present in the references file. If both are found, their positions are used to extract the region of interest for classification and, if the end segment precedes the start segment, to reorient the read by taking its reverse complement. If either segment is absent, the read is given the classification ‘None’ and processing continues to the next read. For reads in which a valid region is found, the extracted sequence is further processed by carrying out a semi-global alignment of each remaining segment sequence in the references file. For each segment, only the single highest-scoring mapping is taken forward, and only if its score exceeds an automatically calculated threshold. Where two alignments overlap, only the higher-scoring one is retained. From these alignments, a classification is generated as a string of hyphen-separated segment IDs in the order in which they are found. A plain ID denotes an alignment in the forward direction, whereas an ID appended with an asterisk denotes an alignment in the reverse direction. For example, the classification ‘start–seg1–seg2*–end’ indicates that the seg1 sequence was found before the reverse complement of the seg2 sequence. Segment sequences should be long enough to be unique, and are typically 20–100 bp long. An overview of the key inputs and the process is shown in Supplementary Fig. 2.

### Computational analysis

Analysis of sequencing data was performed using segment version 1.0.0. Additional computational analyses, numerical simulations of the mathematical model, and all plots were produced using custom Python scripts. These were run using Python version 3.12.9 with matplotlib version 3.10.0, pandas version 2.2.3, numpy version 1.26.4, and scipy version 1.14.1. Visualizations of genetic circuits were generated in SBOL Visual format^72^ using DNAplotlib version 1.0^73^ and paraSBOLv version 1.0^74^. Figures were composed using Affinity Studio version 3.2.2.

### Statistics and reproducibility

Sample sizes were chosen to match typical practices in the field (i.e., at least biological duplicates for DNA sequencing data). No data were excluded from the analyses. The experiments were not randomized. The investigators were not blinded to allocation during experiments and outcome assessment.

### Reporting summary

Further information on research design is available in the Nature Portfolio Reporting Summary linked to this article.

## Supplementary information

Supplementary Information

Description of Additional Supplementary Files

Supplementary Data 1

Supplementary Data 2

Supplementary Data 3

Reporting Summary

Transparent Peer Review file

## Source data

Source Data

## Data Availability

Python scripts for all OT-2 protocols and custom labware definitions for the automated elements of the workflow are included as Supplementary Data 1^75^ and the live repository can be found at: https://github.com/BiocomputeLab/segment-workflow. A description of all the pre-requisite reagents and equipment, plus a summary of all steps involved are provided in Supplementary Note 1. The DNA sequencing data generated in this study have been deposited in the European Nucleotide Archive (ENA) under accession code PRJEB123545 [https://www.ebi.ac.uk/ena/browser/view/ERP203487]. In addition, all sequencing data is available from Zenodo at: https://doi.org/10.5281/zenodo.15316600/. Source Data are provided with this paper containing all data from figures presented in this work and GenBank files for all genetic constructs used. Source data are provided with this paper.

## Code availability

A snapshot of the code repository for the segment tool is included as Supplementary Data 2 and the live repository can be found at: https://github.com/BiocomputeLab/segment. Python code to numerically simulate the genetic recombinase-based cascade is provided as Supplementary Data 3.
