# Supplementary material for: Characterization of recombinase-based genetic parts and circuits using nanopore sequencing
Source: Nat Commun. 2026 Sep 18;17:10969. doi: 10.1038/s41467-026-77605-x (PMC13683369; doi:10.1038/s41467-026-77605-x)
Supplement: Supplementary file 2 [file 41467_2026_77605_MOESM2_ESM.pdf]

Description of Additional Supplementary Files

Supplementary Data 1: Python scripts for all OT-2 protocols and custom labware definitions for the automated elements of the workflow. The live repository can be found at: https://github.com/BiocomputeLab/segment-workflow

Supplementary Data 2: A snapshot of the code repository for the segment tool used in this work. The live repository can be found at: https://github.com/BiocomputeLab/segment

Supplementary Data 3: Python code to numerically simulate the genetic recombinase-based cascade.
