# Supplementary material for: Characterization of recombinase-based genetic parts and circuits using nanopore sequencing
Source: Nat Commun. 2026 Sep 18;17:10969. doi: 10.1038/s41467-026-77605-x (PMC13683369; doi:10.1038/s41467-026-77605-x)
Supplement: Supplementary file 3 [file 41467_2026_77605_MOESM6_ESM.pdf]

nature portfolio

Corresponding author(s): Thomas E. Gorochowski

Last updated by author(s): 10/08/2026

Reporting Summary

Nature Portfolio wishes to improve the reproducibility of the work that we publish. This form provides structure for consistency and transparency in reporting. For further information on Nature Portfolio policies, see our Editorial Policies and the Editorial Policy Checklist.

Statistics

For all statistical analyses, confirm that the following items are present in the figure legend, table legend, main text, or Methods section.

n/a	Confirmed	
☐	☒	The exact sample size (*n*) for each experimental group/condition, given as a discrete number and unit of measurement
☐	☒	A statement on whether measurements were taken from distinct samples or whether the same sample was measured repeatedly
☒	☐	The statistical test(s) used AND whether they are one- or two-sided<br>*Only common tests should be described solely by name; describe more complex techniques in the Methods section.*
☒	☐	A description of all covariates tested
☒	☐	A description of any assumptions or corrections, such as tests of normality and adjustment for multiple comparisons
☐	☒	A full description of the statistical parameters including central tendency (e.g. means) or other basic estimates (e.g. regression coefficient) AND variation (e.g. standard deviation) or associated estimates of uncertainty (e.g. confidence intervals)
☒	☐	For null hypothesis testing, the test statistic (e.g. F, t, r) with confidence intervals, effect sizes, degrees of freedom and P value noted<br>*Give P values as exact values whenever suitable.*
☒	☐	For Bayesian analysis, information on the choice of priors and Markov chain Monte Carlo settings
☒	☐	For hierarchical and complex designs, identification of the appropriate level for tests and full reporting of outcomes
☒	☐	Estimates of effect sizes (e.g. Cohen's *d*, Pearson's *r*), indicating how they were calculated

Our web collection on statistics for biologists contains articles on many of the points above.

Software and code

Policy information about availability of computer code

Data collection	MinKNOW version 21.11.7 was used to collect all DNA sequencing data.
Data analysis	FAST5 sequencing data was basecalled and demultiplexed using guppy version 6.5.7 using the 'dna_r9.4.1_450bps_hac.cfg' high-accuracy model and 'EXP-PBC096' barcode kit to generate sample-specific FASTQ files that could then be used as input for downstream analyses. Analysis of FASTQ files was performed using segment version 1.0.0. Additional computational analyses and all plots were produced using custom Python scripts. These were run using Python version 3.12.9 with matplotlib version 3.10.0, pandas version 2.2.3, numpy version 1.26.4, and scipy version 1.14.1.

For manuscripts utilizing custom algorithms or software that are central to the research but not yet described in published literature, software must be made available to editors and reviewers. We strongly encourage code deposition in a community repository (e.g. GitHub). See the Nature Portfolio guidelines for submitting code & software for further information.

Data

Policy information about availability of data

All manuscripts must include a data availability statement. This statement should provide the following information, where applicable:

- Accession codes, unique identifiers, or web links for publicly available datasets
- A description of any restrictions on data availability
- For clinical datasets or third party data, please ensure that the statement adheres to our policy

Python scripts for all OT-2 protocols and custom labware definitions for the automated elements of the workflow are included as Supplementary Data 1 and the live repository can be found at: https://github.com/BiocomputeLab/segment-workflow. A description of all the pre-requisite reagents/equipment plus a summary of all steps involved are provided in Supplementary Note 1. The DNA sequencing data generated in this study have been deposited in the European Nucleotide Archive (ENA) under accession code PRJEB123545 [https://www.ebi.ac.uk/ena/browser/view/ERP203487]. In addition, all sequencing data is available from Zenodo at: https://doi.org/10.5281/zenodo.15316600/. Source Data are provided with this paper containing all data from figures presented in this work and GenBank files for all genetic constructs used. A snapshot of the code repository for the segment tool is included as Supplementary Data 2 and the live repository can be found at: https://github.com/BiocomputeLab/segment. Python code to numerically simulate the genetic recombinase-based cascade is provided as Supplementary Data 3.

Research involving human participants, their data, or biological material

Policy information about studies with human participants or human data. See also policy information about sex, gender (identity/presentation), and sexual orientation and race, ethnicity and racism.

Reporting on sex and gender	N/A
Reporting on race, ethnicity, or other socially relevant groupings	N/A
Population characteristics	N/A
Recruitment	N/A
Ethics oversight	N/A

Note that full information on the approval of the study protocol must also be provided in the manuscript.

Field-specific reporting

Please select the one below that is the best fit for your research. If you are not sure, read the appropriate sections before making your selection.

☒ Life sciences ☐ Behavioural & social sciences ☐ Ecological, evolutionary & environmental sciences

For a reference copy of the document with all sections, see nature.com/documents/nr-reporting-summary-flat.pdf

Life sciences study design

All studies must disclose on these points even when the disclosure is negative.

Sample size	Sample sizes were chosen to match typical practices in the field (i.e., at least biological duplicates for DNA sequencing data).
Data exclusions	No data were excluded from the analyses.
Replication	Biological replicates were performed for all experiments (n = 2 or 3 for all experiments).
Randomization	Randomization is not relevant to this study as all constructs tested were sequenced.
Blinding	Blinding is not relevant for this study as all constructs tested were tested.

Reporting for specific materials, systems and methods

We require information from authors about some types of materials, experimental systems and methods used in many studies. Here, indicate whether each material, system or method listed is relevant to your study. If you are not sure if a list item applies to your research, read the appropriate section before selecting a response.

Materials & experimental systems

n/a	Involved in the study
☒	☐ Antibodies
☒	☐ Eukaryotic cell lines
☒	☐ Palaeontology and archaeology
☒	☐ Animals and other organisms
☒	☐ Clinical data
☒	☐ Dual use research of concern
☐	☒ Plants

Methods

n/a	Involved in the study
☒	☐ ChIP-seq
☒	☐ Flow cytometry
☒	☐ MRI-based neuroimaging

Dual use research of concern

Policy information about dual use research of concern

Hazards

Could the accidental, deliberate or reckless misuse of agents or technologies generated in the work, or the application of information presented in the manuscript, pose a threat to:

No	Yes
☒	☐ Public health
☒	☐ National security
☒	☐ Crops and/or livestock
☒	☐ Ecosystems
☒	☐ Any other significant area

Experiments of concern

Does the work involve any of these experiments of concern:

No	Yes
☒	☐ Demonstrate how to render a vaccine ineffective
☒	☐ Confer resistance to therapeutically useful antibiotics or antiviral agents
☒	☐ Enhance the virulence of a pathogen or render a nonpathogen virulent
☒	☐ Increase transmissibility of a pathogen
☒	☐ Alter the host range of a pathogen
☒	☐ Enable evasion of diagnostic/detection modalities
☒	☐ Enable the weaponization of a biological agent or toxin
☒	☐ Any other potentially harmful combination of experiments and agents

Plants

Seed stocks	Arabidopsis thaliana columbia (Col-0) ecotype seeds were obtained from Jose Dinneny at Stanford University.
Novel plant genotypes	Transgenic plant lines were generated through Agrobacterium-mediated floral dip transformation. Six independent lines were generated and analyzed for T-DNA segregation to identify homozygous lines containing a single T-DNA insertion locus.
Authentication	Transgenic seeds were identified using a ubiquitously expressed mCherry marker and a fluorescence microscope. Fluorescent seeds were germinated and seedlings were imaged five days post germination to confirm expression of the fluorescent protein. Homozygous lines containing a single T-DNA insertion locus were identified through segregation analysis of T2 and T3 seeds. Only single insertion transgenic lines were used for the experiments described in the paper.
