# Supplementary material for: Characterization of recombinase-based genetic parts and circuits using nanopore sequencing
Source: Nat Commun. 2026 Sep 18;17:10969. doi: 10.1038/s41467-026-77605-x (PMC13683369; doi:10.1038/s41467-026-77605-x)
Supplement: Supplementary file 4 [file 41467_2026_77605_MOESM7_ESM.pdf]

nature portfolio

Peer Review File

Characterization of recombinase-based genetic parts and circuits using nanopore sequencing

Corresponding Author: Professor Thomas Gorochowski

This file contains all reviewer reports in order by version, followed by all author rebuttals in order by version.

Version 0:

Reviewer comments:

Reviewer #1

(Remarks to the Author)
Greco et al. present a semi-automated pipeline that couples liquid handling robots, ONT sequencing, and a computational analysis suite for the characterisation of recombinase-based genetic circuits. The authors used this pipeline to characterise the kinetics of six recombinases in E. coli, monitor a dual-plasmid recombinase cascade, and quantify the spatial activation of a recombinase switch in A. taliana.
The authors have done an excellent job of keeping everything open-source and documented, and because nothing in the hardware, barcoding, or software is intrinsically tied to serine integrases, I am sure this work will be very useful to the synthetic biology community well beyond the specific application reported in the paper.
Although this study is scientifically solid and experiments well conducted, I have a few comments that I think should be addressed before publication.

Major corrections
• Flip percentages are derived solely from nanopore sequencing data. Because one of the key achievements of the paper is the establishment of a long-read sequencing pipeline to assess recombinase activity, an orthogonal validation method of the flip percentages would give confidence in the numerical accuracy of the pipeline. I believe validating just one of the panels in figure 2b would be sufficient. As a validation method, I suggest a ddPCR or qPCR experiment with probes on the attb/attP junction, but the authors might think of an alternative/better approach.
• The authors quote a per-sample reagent cost of £ 5.89 excluding flow cells and ligation sequencing kit. Since the authors do already provide a cost estimate, I would ask them to provide a comprehensive one. Because the cost of the sequencing depends on how many barcoded samples are run on a single flongle, providing a per sample cost curve versus multiplexing depth (e.g. 24, 48, 96, or more samples) would make the cost analysis more transparent to the reader.

Minor corrections
• In Figure 2d, overlaying 50 bp and 600–900 bp curves in the same graph would make an easier comparison.
• The caption of Figure 2 does not specify the number of biological replicates. Although these can be inferred from the graphs, as the authors displays data points relating to each replicate, explicitly stating the number of replicates in the caption would make it easier to the reader.
• In many instances in the supplementary information “µL” is reported as “uL”.

(Remarks on code availability)

Reviewer #2

(Remarks to the Author)
Greco et al. present a semi-automated workflow for characterizing recombinase-based genetic circuits using nanopore sequencing. The authors developed an integrated experimental and computational pipeline combining low-cost liquid handling robotics and multiplexed long-read sequencing to monitor recombinase circuit states over time. They demonstrate the workflow's capabilities by characterizing six recombinase enzymes, tracking cascade dynamics, and mapping spatial activation of a recombinase switch in plant roots.

This is a promising technological development that addresses a real need in the synthetic biology community. Compared to fluorescence-based approaches, this method avoids the need to express heterogeneous proteins, which imposes a burden on cellular resource, and is difficult to calibrate under changing conditions or multiplex at scale. However, I feel the paper does not fully explore its potential, and its current form reads more like a proof of concept than a comprehensive demonstration. A critical question is how this method fundamentally differs from traditional fluorescence-based approaches. In the abstract, the authors state that their approach "...observe structural changes at nucleotide resolution, and unravel the internal workings of complex multi-state circuits," which is intriguing. It would be helpful if the authors could provide additional justification for these claims. I provide the following comments with the hope that they will help strengthen the manuscript.

Major comments:
- The authors use the tool segment to map sequencing reads to a limited number of predefined circuit states, each defined as combinations of short DNA fragments. My concern is that this approach may not capture the full complexity of recombinase circuits. How could it be extended to identify rare intermediates or failure modes that fluorescence-based markers might miss?

- Figure 2c is interesting. My questions here are twofold: (1) How did the authors measure off-target effects—did they map reads to all 2^11 possible combinations? (2) What exactly happens in these off-target reads? For example, when the authors mention off-target activity for Int4 across multiple recognition sites (Int9–Int12), does this imply Int4 flips the entire Int9 site or only part of it? Clarifying and illustrating these off-target scenarios would help demonstrate the method's strength in characterizing these circuits at single-nucleotide resolution.

- The manuscript would benefit from a systematic benchmarking analysis, such as calibrating sequencing outputs against steady-state fluorescence measurements. I also suggest that the authors discuss potential strategies to address PCR-amplification bias in the discussion section.

- In Figure 3, only about 50% of cells switched to the intended state by the end of the experiment (8h). This is in contrast with the near-perfect performance shown in Figure 2. Could the authors provide potential explanations for this result? I noticed that the fraction of DNA flipped by Int5 is lower in Figure 3 than in Figure 2. The target memory array in Figure 3 is on a higher-copy ColE1 plasmid (versus the p15A backbone in Figure 2). Could the elevated DNA copy number be titrating Int5, reducing the effective recombinase-to-target ratio?

- On a related note, is it possible to use a modeling approach to capture the dynamics of the cascade circuit in Figure 3?

Minor comments
- I would recommend the authors to include further details about the segment tool in the methods part, including the bioinformatics tools/packages used.
- Line 117: "performed an additional (experiment) with samples..."
- Figure 3c is not mentioned in the main text.

(Remarks on code availability)

Version 1:

Reviewer comments:

Reviewer #1

(Remarks to the Author)
I am satisfied with the improvements, and I thank the authors for acting on my comments. I have no outstanding comments and recommend publication

(Remarks on code availability)

Reviewer #2

(Remarks to the Author)
The authors have addressed my concerns and the manuscript has been substantially improved. I have one minor comment regarding lines 240–249. The discussion appropriately identifies several factors that may slow recombination. If flipping is slow, unconverted plasmids may persist and continue to replicate during the experimental period, potentially limiting the extent of conversion. Thus, the observed dynamics may depend on the relative rates of recombinase-mediated flipping and plasmid replication or dilution, rather than on the flipping rate alone. Based on the green curve in Fig. 4d, these processes appear to occur on broadly similar timescales.

(Remarks on code availability)

Open Access This Peer Review File is licensed under a Creative Commons Attribution 4.0 International License, which permits use, sharing, adaptation, distribution and reproduction in any medium or format, as long as you give appropriate credit to the original author(s) and the source, provide a link to the Creative Commons license, and indicate if changes were made.
In cases where reviewers are anonymous, credit should be given to 'Anonymous Referee' and the source.
The images or other third party material in this Peer Review File are included in the article's Creative Commons license, unless indicated otherwise in a credit line to the material. If material is not included in the article's Creative Commons license and your intended use is not permitted by statutory regulation or exceeds the permitted use, you will need to obtain permission directly from the copyright holder.
To view a copy of this license, visit https://creativecommons.org/licenses/by/4.0/

Response to reviewers (NCOMMS-25-52996-T)

Find below our response (in blue) to all the reviewers' comments. We believe that our updates to the manuscript address all their concerns and we are thankful for their feedback. This has greatly improved the piece. We have also carefully reviewed the entire manuscript and made minor changes throughout to improve readability. In the revised manuscript, sentences containing edits have been entirely highlighted in red to aid reviewing.

Reviewer #1:

Greco et al. present a semi-automated pipeline that couples liquid handling robots, ONT sequencing, and a computational analysis suite for the characterisation of recombinase-based genetic circuits. The authors used this pipeline to characterise the kinetics of six recombinases in E. coli, monitor a dual-plasmid recombinase cascade, and quantify the spatial activation of a recombinase switch in A. taliana. The authors have done an excellent job of keeping everything open-source and documented, and because nothing in the hardware, barcoding, or software is intrinsically tied to serine integrases, I am sure this work will be very useful to the synthetic biology community well beyond the specific application reported in the paper. Although this study is scientifically solid and experiments well conducted, I have a few comments that I think should be addressed before publication.

We thank the reviewer for their thoughtful consideration of our work and positive feeling as to its suitability for publication. Below we have aimed to address all their concerns and queries raised.

Major corrections

• Flip percentages are derived solely from nanopore sequencing data. Because one of the key achievements of the paper is the establishment of a long-read sequencing pipeline to assess recombinase activity, an orthogonal validation method of the flip percentages would give confidence in the numerical accuracy of the pipeline. I believe validating just one of the panels in figure 2b would be sufficient. As a validation method, I suggest a ddPCR or qPCR experiment with probes on the attb/attP junction, but the authors might think of an alternative/better approach.

This is an excellent point. To address the reviewer's comment, rather than using another method, where additional biases may be present, we instead chose to set up an experiment in which the ground truth (in terms of the proportions of states in the system) was known. We achieved this by generating memory array plasmids in two different configurations, transforming these into separate populations of cells that were unable to further modify the memory array, and then artificially creating mixed cultures with known proportions of each. These experiments showed that our workflow can accurately recover the known proportions of states in a population of cells. These new results have been included as a new figure panel (Figure 2c) and in the updated Results section of the revision:

"*To assess the accuracy of the measurements from the workflow, we performed further experiments with populations of cells containing known fractions of the memory array where a specific set of sites had been flipped. To do this, we generated memory array plasmids in two different states. The first state was the original plasmid with all sites unflipped. The second state was the memory array after exposure to Int2, Int5, Int7 and Int8 such that the cognate sites for these recombinases had become fully flipped. Plasmids in this second state were then purified and sequence verified to ensure they contained the correct configuration. Finally, two separate populations of cells were transformed. The first with the original unflipped memory array, and the second with the purified memory array with a semi-flipped configuration. Importantly, cells from each population contained no recombinase expressing plasmid (i.e., pIntX), ensuring no further changes would be made to the state of the memory array. These two populations were then separately grown, their OD600 measured and varying proportions of the populations mixed to generate expected factions of unflipped and flipped memory arrays. We chose 0, 2.5, 5, 50 and 100 flipped percentages to be able to assess the sensitivity*

*of the workflow to small differences, while also testing the full range of possible values. Comparing the expected flipped percentage of the Int2, Int5, Int7 and Int8 sites to those measured, we saw a strong correlation with R^2 > 0.975 for each site (**Figure 2c**). Also, as expected, virtually no flipping of other recombinase sites was seen, with only two samples showing very low levels of incorrect flipped states for three of the recombinase sites (<0.002% for Int3, Int4 and Int12). These results suggest that the workflow is accurate and sensitive enough to measure even small changes (<10%) in the state of recombinase sites within the memory array.***"**

• The authors quote a per-sample reagent cost of £5.89 excluding flow cells and ligation sequencing kit. Since the authors do already provide a cost estimate, I would ask them to provide a comprehensive one. Because the cost of the sequencing depends on how many barcoded samples are run on a single flongle, providing a per sample cost curve versus multiplexing depth (e.g. 24, 48, 96, or more samples) would make the cost analysis more transparent to the reader.

This is a good point. In the revision, we have added additional information covering the sequencing cost per sample for 24–192 samples when using standard MinION flow cells (i.e., FLO-MIN114). This is under the assumption of full UK prices for only a single flow cell. These costs reduce significantly (>30%) when bulk purchasing prices for flow cells are considered:

"*Flow cell and sequencing library preparation costs per sample scale based on the level of multiplexing that is performed. For 24, 48, 96 and 192 samples per MinION flow cell, the cost per sample is approximately £32.84, £16.42, £8.21 and £4.10, respectively, with these costs under the assumption of full UK prices. These costs reduce significantly (>30%) if bulk purchasing of flow cells is performed as these are where most of the sequencing costs lie.***"**

Minor corrections

• In Figure 2d, overlaying 50 bp and 600–900 bp curves in the same graph would make an easier comparison.

As recommended by the reviewer, we have updated Figure 2d to include a dotted black line corresponding to the 50 bp long spacer data (taken from Figure 2b).

• The caption of Figure 2 does not specify the number of biological replicates. Although these can be inferred from the graphs, as the authors displays data points relating to each replicate, explicitly stating the number of replicates in the caption would make it easier to the reader.

We have updated the caption for Figure 2 to state the number of biological replicates for each relevant panel: "*Black circles and crosses are individual measurements from the three biological replicates for samples taken at differing time points: 0, 40, 80, 120, 150, 180, 210, 240, and 360 min, and 20, 30, 40, 50, 60, 70, 80, 90, 100, and 160 min, respectively.***" and "***Solid circles are individual measurements from the three biological replicates for samples taken at differing time points.***". We also noticed this was not mentioned in Figure 4d and so updated the caption for that figure too: "***Activation of the genetic switch in different sections of the root. Values for biological replicates (two for samples near leaves and from the root tip, and three for the lateral roots) of the experiment are shown by white-filled circles.***"**

• In many instances in the supplementary information "µL" is reported as "uL".

We apologise for these typos. We have carefully checked through the entire manuscript and corrected issues where necessary.

Reviewer #2:

Greco et al. present a semi-automated workflow for characterizing recombinase-based genetic circuits using nanopore sequencing. The authors developed an integrated experimental and computational pipeline combining low-cost liquid handling robotics and multiplexed long-read sequencing to monitor recombinase circuit states over time. They demonstrate the workflow's capabilities by characterizing six recombinase enzymes, tracking cascade dynamics, and mapping spatial activation of a recombinase switch in plant roots.

This is a promising technological development that addresses a real need in the synthetic biology community. Compared to fluorescence-based approaches, this method avoids the need to express heterogeneous proteins, which imposes a burden on cellular resource, and is difficult to calibrate under changing conditions or multiplex at scale. However, I feel the paper does not fully explore its potential, and its current form reads more like a proof of concept than a comprehensive demonstration. A critical question is how this method fundamentally differs from traditional fluorescence-based approaches. In the abstract, the authors state that their approach "...observe structural changes at nucleotide resolution, and unravel the internal workings of complex multi-state circuits," which is intriguing. It would be helpful if the authors could provide additional justification for these claims. I provide the following comments with the hope that they will help strengthen the manuscript.

We thank the reviewer for their detailed assessment and suggestions for strengthening our work. Below we have attempted to address all their comments.

Major comments:

- The authors use the tool segment to map sequencing reads to a limited number of predefined circuit states, each defined as combinations of short DNA fragments. My concern is that this approach may not capture the full complexity of recombinase circuits. How could it be extended to identify rare intermediates or failure modes that fluorescence-based markers might miss?

This is a great question that we believe in part arises from a lack of elaboration on how the generation of segment classifications can be used. Firstly, the reviewer is correct that our segment analysis will not capture every type of change in a circuit. By abstracting a circuit's state into the order and orientation of a sequence of segments, we necessarily discard some nucleotide-level detail. In return, this greatly simplifies interpretation of the sequencing data: it gives clear insight into the critical components of a circuit (i.e., regions that might encode promoters, coding sequences, terminators, etc.) and into how their positions and orientations vary across the circuit. This captures most of the key information needed to assess whether a circuit is working and, when it is not, how it has broken (e.g., a segment flipping when it should not).

Secondly, the segments a user defines often tile large regions of the circuit (see, for example, the sections on the memory array and the cascade circuit). This means that although small nucleotide changes fall below our resolution, larger structural failures do not. Consider a circuit that becomes unstable due to part reuse, causing a section to loop out. Such an event removes an entire segment, and so appears in our analysis as an unexpected state in which that segment is missing altogether. Duplications are detected in the same way, appearing as states with repeated segments. Because the segment classifications also record the physical order of elements, the root cause of these events is then straightforward to identify. This ability to handle circuits whose states grow or shrink is a key benefit of our approach. It is not possible with full reference-based methods, because in these cases the state space of the circuit is effectively infinite.

Finally, regarding failure modes that fluorescence-based markers might miss: a more detailed analysis of off-target effects in the memory array (see our response to the next comment) showed that even when the correct cognate integrase site is flipped, other portions of the array can be affected by off-target activity. No fluorescence-based assay would be able to capture such changes in situ with no modifications to the circuit.

We have updated the Discussion to better explain some of these benefits:

"*A major advantage of our approach for classifying read configurations using short, user-defined "segments" is that it removes the need to generate reference sequences for every possible circuit configuration. Mapping-based approaches, which rely on tools such as BLASTn or minimap2, require a full reference sequence in order to find the most probable alignment for each read. This is feasible for the genetic memory array considered here (**Figure 2**), which has 2048 possible configurations. As circuit complexity grows, however, enumerating every reference sequence quickly becomes intractable due to memory constraints. A recombinase circuit with just 30 independently invertible regions, for example, already has over 1 billion possible configurations, and this number grows far more rapidly when overlapping inversions, excisions and insertions are possible. The problem becomes insurmountable for larger systems such as the engineered Sc2.0 genome [43, 44]. When complete, this genome will contain approximately 3,000 DNA segments flanked by loxPsym sites that enable large-scale chromosome rearrangements. Because chromosomes can expand during recombination procedures like SCRaMbLE [44], the number of possible configurations is unbounded, and reference-based approaches cannot be applied at all.*

*By analyzing the order and orientation of key segment sequences instead, we can classify reads efficiently without any complete reference for the system. The resulting data also remain highly interpretable, making it possible to pinpoint the root cause of failures such as unexpected duplications and deletions arising from circuit instability [45, 46]. In addition, because the segments used to characterize a construct's state typically tile the entire circuit, it is straightforward to zoom in on the most relevant region of interest and extract its sequences for deeper analysis. This is precisely how we revealed the off-target attB/attP sites responsible for the unexpected flipping of a large portion of the memory array (Int9–Int12) during Int4 expression (**Figure 3**). Furthermore, many genetic parts and circuits, including recombinases, self-cleaving ribozymes, transcriptional terminators and introns, work by altering the architecture (i.e., the presence, order and orientation of sub-sequences) of DNA and RNA molecules. Our segment-based approach should therefore prove effective across a wide range of other challenging characterization tasks in synthetic biology.***"**

- Figure 2c is interesting. My questions here are twofold: (1) How did the authors measure off-target effects—did they map reads to all 2^11 possible combinations? (2) What exactly happens in these off-target reads? For example, when the authors mention off-target activity for Int4 across multiple recognition sites (Int9–Int12), does this imply Int4 flips the entire Int9 site or only part of it? Clarifying and illustrating these off-target scenarios would help demonstrate the method's strength in characterizing these circuits at single-nucleotide resolution.

We apologise for not explaining the methodologies clearly enough in the original submission. Regarding the measurement of off-target effects, we looked for flipped segments in the memory register that did not correspond to the known recognition site for the recombinase being expressed. This allows us to capture any of the possible 2^{11} different configurations that the register could take, without the need to explicitly create references for each. We also agree that while our method clearly captures nucleotide resolution information, we had not made best use of this information in the original submission. To address this, as suggested by the reviewer, we have substantially extended our off-target analysis to assess precisely where off-targets cuts take place and serendipitously uncover novel recombination sites for Int4 with a low affinity in the memory array. None of this would have been possible using standard fluorescence-based approaches.

"*More detailed analysis of the sequencing data from the memory array showed that all recombinases precisely targeted their cognate sites, with only minor off-target activity for Int4 across numerous recognition sites (Int9–Int12), Int2–Int4 weakly recognizing Int8 sites, and some cross-reactivity between Int5 and Int7, Int2 and Int8, and Int3 and Int8, respectively. In all cases, off-target activity was typically very rare with <0.1% of off-target regions flipped after 2 hours 40 mins (**Figure 2e**). In all cases, this off-target activity related to <10 reads per interaction and in most cases was no more than 1 or 2 reads across multiple biological replicates. It is possible that some of the off-target activity occurred due to incorrect barcode classification during the sequencing step [33]. For example, a read from a sample where Int8 is expression, incorrectly being classified as a read for a sample where Int2 is expressed. In this scenario, given the rarity of barcode misclassifications, we would expect only the off-target site to be flipped. Further analysis of all reads containing off-target activity showed this to be the case, with 83% of a total of 29 reads showing off-target activity across all integrases being*

potentially prone to barcode missclassification. However, we still found 2 reads in which both the correct site was flipped in addition to an off-target site where barcode misclassification is unlikely to have played a role, and this possible error could not account for the additional 3 reads across two biological replicates showing off-target activity for the entire Int9–Int12 region when expressing Int4, as none of those off-target sites had their cognate integrase expressed in any of the samples.

*A further benefit of long-read sequencing for characterization is its ability to provide nucleotide-resolution insight. To demonstrate this ability, we collected reads in which both an expected (cognate) site and an additional off-target site were flipped. These reads were grouped according to the integrase expressed in each sample, and comparisons were then made to infer the precise points at which DNA had been cut at the off-target sites. This allowed us to infer sequences for which each integrase had an affinity, even when these were not its cognate attB/attP sites (**Figure 3a**).*

*For Int2 and Int8 expression, a single read from each sample contained both the Int2 and Int8 spacer sequences flipped (**Figure 3a,b**). Analysis of the off-target activity in both cases showed that the cut site matched the expected cut site for that sequence and its cognate integrase (i.e., for Int2 expression, the Int8 attB and attP sites were cut precisely where Int8 would be expected to cut). As only a single read out of more than 100,000 showed this pattern in each case, this activity is clearly very rare. More interestingly, for Int4 expression we observed flipping of a region whose inferred attB and attP sites did not correspond to any known site for any of the integrases considered in this study (**Figure 3c**). We classified three such reads across two biological replicates, suggesting that this activity was more common than other off-target activity observed and is unlikely to result from sequencing artifacts such as barcode misclassification. The flipped region was 592 bp long, beginning 1 bp before the end of the Int9 attB site and 2 bp from the end of the Int12 attP site.*

*Finally, we used our ability to pinpoint the bases where recombination occurs to also extract consensus cut sites for both the attP and attB sequences across all the integrases studied. As expected, we found that these matched cut sites previously documented in the literature (**Supplementary Table 4**) [21].***"**

- The manuscript would benefit from a systematic benchmarking analysis, such as calibrating sequencing outputs against steady-state fluorescence measurements. I also suggest that the authors discuss potential strategies to address PCR-amplification bias in the discussion section.

This is an excellent suggestion and the recommendation for a calibration of the method was also raised by Reviewer 1. As elaborated on above in our response to Reviewer 1 (first comment), we have now performed an experiment using a known ground truth to allow for expected and measured flipped fractions of the memory array to be properly compared. These new findings have been added to the Results section of the revision and as a new panel in Figure 2:

"*To assess the accuracy of the measurements from the workflow, we performed further experiments with populations of cells containing known fractions of the memory array where a specific set of sites had been flipped. To do this, we generated memory array plasmids in two different states. The first state was the original plasmid with all sites unflipped. The second state was the memory array after exposure to Int2, Int5, Int7 and Int8 such that the cognate sites for these recombinases had become fully flipped. Plasmids in this second state were then purified and sequence verified to ensure they contained the correct configuration. Finally, two separate populations of cells were transformed. The first with the original unflipped memory array, and the second with the purified memory array with a semi-flipped configuration. Importantly, cells from each population contained no recombinase expressing plasmid (i.e., pIntX), ensuring no further changes would be made to the state of the memory array. These two populations were then separately grown, their OD600 measured and varying proportions of the populations mixed to generate expected factions of unflipped and flipped memory arrays. We chose mixtures containing 0%, 2.5%, 5%, 50% and 100% of flipped cells to be able to assess the sensitivity of the workflow to small differences, while also testing the full range of possible values. Comparing the expected flipped percentage of the Int2, Int5, Int7 and Int8 sites to those measured, we saw a strong correlation with $R^2 > 0.975$ for each site (**Figure 2c**). Also, as expected, virtually no flipping of other recombinase sites was seen, with only two samples showing very low levels of incorrect flipped states for three of the recombinase sites (<0.002% for Int3, Int4 and Int12). These results suggest that the workflow*

is accurate and sensitive enough to measure even small changes (<10%) in the state of recombinase sites within the memory array."

In addition, and as suggested by the reviewer, we have added several new paragraphs to the Discussion section to elaborate on how PCR bias may affect the methodology, how its effect can be reduced, and how alternative sequencing library preparation methods (e.g., rapid transposon-based approaches) can be used to further mitigate issues for challenging use cases:

"*A second challenge is that our reliance on PCR to both target and amplify the relevant regions of a recombinase circuit could, in principle, distort the measured relative abundances of different circuit states. Quantitative PCR from a mixed pool of templates is well known to be susceptible to preferential amplification, whereby the composition of the final amplicon pool no longer faithfully reflects the input [46, 47]. It is therefore worth considering the extent to which such biases might affect our measurements and where they are most likely to arise.*

One of the dominant sources of bias in multi-template PCR stems from differences in primer–template binding kinetics. When the templates in a reaction differ across their primer-binding sites (e.g., through sequence divergence or the use of degenerate primers) they anneal to the primers with different efficiencies and binding energies, with more efficiently primed templates become progressively over-represented each cycle [47, 48]. Even a single primer–template mismatch can substantially skew the relative amplification of otherwise similar templates [49, 50]. Our workflow is designed to avoid this class of bias entirely. Because recombination acts on the interior of the circuit while leaving the flanking regions untouched, the same pair of tailing primers bind the same invariant sequences irrespective of circuit state, so every state is primed with identical kinetics and no state is favored at the priming step. The subsequent barcoding PCR likewise primes from the common tails shared by all amplicons, and because a given barcode is applied per sample rather than per state, it cannot distort the relative proportions of states measured within that sample.

*The bias that our design does not eliminate arises later, during elongation and amplification, and is driven by properties of the amplicon itself (e.g., base composition and length). Templates with extreme GC content are amplified less efficiently, because GC-rich duplexes are harder to denature and can form secondary structures that affect polymerase progression, while very AT-rich regions are similarly prone to under-representation. Due to these effects, PCR during library preparation has been identified as the principal source of this base-composition bias in sequencing data [51, 52]. Length introduces a second effect with shorter amplicons amplified more efficiently than longer ones and, when considering a mixed reaction, out-compete longer templates causing shorter states to become over-represented. For example, a size-dependent template competition has been shown to strongly suppress the recovery of longer fragments in length-polymorphic genotyping and metabarcoding [53–55]. Importantly, these effects are negligible for the circuits characterized here. All of the recombination events we measure, i.e., the flipping of segments in the memory array (**Figure 2**), the inversion of recombinase and reporter coding sequences in the cascade (**Figure 3**), and the flipping of the promoter in the plant switch (**Figure 4**), are inversions that leave the length and overall base composition of the amplified region unchanged between states. The two orientations of an inverted segment should therefore be amplified with near-identical efficiency, and the strong agreement we observe between expected and measured flipped fractions across the full potential range is consistent with this expectation. However, composition- and length-dependent bias becomes a genuine concern for circuits in which recombination alters the amplicon itself. For example, circuits employing excisions or insertions that shorten or lengthen the region between the tailing primers, where a shorter state could become over-represented relative to a longer one.*

Where such length or composition differences are unavoidable, several strategies can reduce or correct the residual bias. Because bias accrues disproportionately in the later, more competitive cycles of the reaction, minimizing the cycle number, increasing reaction volumes, and pooling replicate reactions all help to keep the product distribution closer to the template distribution [46, 47]. Incorporating unique molecular identifiers into the tailing primers would allow individual template molecules to be counted after amplification, correcting for uneven copying directly at the analysis stage [56]. More fundamentally, the amplification step can be reduced or removed altogether by enriching the target DNA through hybridization capture with biotinylated probes [57] or Cas9-directed cleavage [58], and by attaching barcoded sequencing adapters through direct ligation or

transposase-based tagmentation (e.g., using rapid barcoding kits for sequencing library preparation) rather than a second PCR [59]. These amplification-free routes are particularly attractive for circuits whose states differ substantially in length, and represent a natural elaboration of our workflow for such use cases."

- In Figure 3, only about 50% of cells switched to the intended state by the end of the experiment (8h). This is in contrast with the near-perfect performance shown in Figure 2. Could the authors provide potential explanations for this result? I noticed that the fraction of DNA flipped by Int5 is lower in Figure 3 than in Figure 2. The target memory array in Figure 3 is on a higher-copy ColE1 plasmid (versus the p15A backbone in Figure 2). Could the elevated DNA copy number be titrating Int5, reducing the effective recombinase-to-target ratio?

This is an interesting observation. Several differences between the single-recombinase characterisation experiments and the cascade circuit could account for the switching dynamics observed. The reviewer is correct that the higher copy number of the ColE1 plasmid would lead to a lower relative concentration of Int2 at its target recombination sites, slowing the flipping rate. In addition, the lag we observe between activations is a further potential cause: time is needed for a recombinase that has recently been switched into an active state to be expressed to a level sufficient for efficient recombination at the next step in the cascade. The context of the recombination sites also differs substantially, with the distance between cognate sites being much greater than the 50 bp separating them in the memory array. Together, these factors are likely to account for the much slower dynamics. This is further supported by a model we developed to address the reviewer's next comment, which shows that not all of the final GFP coding region has been flipped after 8 hours (50% flipping occurs at around 6.5 hours). To make these factors clear, we have expanded this section of the Results to highlight the differences in context that may account for the slower dynamics observed for the cascade:

"*Analysis showed a clear propagation of inversions in the coding sequences of the recombinases and GFP over time that corresponded to the sequential expression of each recombinase in the cascade (**Figure 3d**). The changes observed also qualitatively matched the response seen by our model. Similar to the characterization experiments of the recombinases in isolation, we saw increased rates of sequence inversion by Int5 compared to Int7. Interestingly, the dynamics of the cascade circuit are notably slower than those observed in the single-recombinase characterization experiments. This difference could stem from several sources. First, the initial characterization experiments expressed the recombinase from a higher-copy plasmid (ColE1 origin, ~20 copies per cell) than the target memory array (p15A origin, ~10 copies per cell), whereas many of the steps in the cascade encoded the recombinase expression cassette and its target DNA sites at identical copy numbers per cell (e.g., Int2 and Int7), effectively reducing the recombinase concentration per site and slowing the flipping rate. Second, switching each recombinase in sequence takes additional time, as expression of a newly activated recombinase must reach a sufficiently high concentration to efficiently trigger the next recombinase in the cascade. Finally, the context of the recombination sites also differs, with the distance between cognate sites being much greater than the 50 bp separating them in the memory array, potentially slowing the rate at which recombination proceeds.***"**

- On a related note, is it possible to use a modeling approach to capture the dynamics of the cascade circuit in Figure 3?

This is an excellent suggestion. As recommended by the reviewer, we developed a mathematical model to capture the dynamics of the recombinase-based cascade circuit, and we have included results from this model in the revised Results section and as an additional panel in Figure 3. Details of the model's derivation and its assumptions are provided in the Methods section. Using biologically feasible parameter values, the simulations show good correspondence with the dynamics observed from the direct sequencing measurements. This supports the conclusion that our approach accurately monitors the full internal state of the circuit, and again highlights our ability to provide information that no fluorescence-based assay could achieve:

"*To explore how this circuit might behave, we used the characterization data previously collected for the recombinases in isolation (**Figure 2**) to develop a mathematical model of the circuit's dynamics (**Methods**). The model was based on ordinary differential equations (ODEs), with variables capturing the fraction of flipped recombinase and GFP coding regions across the population, as well as the concentrations of the expressed recombinase and GFP proteins. The variables describing the flipped state of the recombinase and GFP coding regions corresponded directly to measurements that could be made using our sequencing approach. Where possible, we used parameters measured in our own experiments (i.e., from **Figure 2b**), and biologically feasible values otherwise.*

*Numerical simulations of the model showed the expected sequential expression of the recombinases as their coding sequences were flipped, until the final output gene (gfp) was activated (**Figure 3b**). The rate of flipping for each recombinase slowed over time because of the reduced concentration of unflipped substrate arising from the irreversibility of the process (e.g., see the Int5 and Int7-gfp states). We also observed a delay of ~30 min between flipping of the GFP coding region and notable increases in GFP protein expression, reflecting the time required to produce the protein. Note that our model did not account for maturation of GFP (~25 min for many GFP variants [31]), which is required for it to be monitored by fluorescence. Therefore, experiments would likely observe even longer delays between inversion of the GFP coding region and a measurable fluorescence output from the circuit.***"**

Minor comments

- I would recommend the authors to include further details about the segment tool in the methods part, including the bioinformatics tools/packages used.

We apologise for having not included sufficient information about how the segment tool works. As suggested, we have added a new section to the Methods to elaborate on software dependencies, how it is used, and the steps performed during the segment classification of the sequencing reads:

"*We developed a tool called `segment' to classify the configuration of the long nanopore reads from our experiments. The tool is written in the Rust (edition 2021) programming language and depends on the `bio' version 1.0 and `clap' version 4.5.9 crates for efficient semi-global alignment of short segment sequences and for command-line argument handling, respectively. The tool is run through a command-line interface (CLI) and takes three inputs: a set of reference sequences (the segments to search for) in multi-FASTA format; the minimum and maximum length of a valid read (e.g., to filter out short contaminants and long concatemer reads); and the reads to be processed in FASTQ format. Each read is then processed to generate its segment classification. Processing begins by searching the read for the special `start' and `end' segment sequences, which must always be present in the references file. If both are found, their positions are used to extract the region of interest for classification and, if the end segment precedes the start segment, to reorient the read by taking its reverse complement. If either segment is absent, the read is given the classification `None' and processing continues to the next read. For reads in which a valid region is found, the extracted sequence is further processed by carrying out a semi-global alignment of each remaining segment sequence in the references file. For each segment, only the single highest-scoring mapping is taken forward, and only if its score exceeds an automatically calculated threshold. Where two alignments overlap, only the higher-scoring one is retained. From these alignments, a classification is generated as a string of hyphen-separated segment IDs in the order in which they are found. A plain ID denotes an alignment in the forward direction, whereas an ID appended with an asterisk denotes an alignment in the reverse direction. For example, the classification `start–seg1–seg2*–end' indicates that the seg1 sequence was found before the reverse complement of the seg2 sequence. Segment sequences should be long enough to be unique, and are typically 20–100 bp long. An overview of the key inputs and the process is shown in **Supplementary Figure 2**, and details of how to access the tool can be found in the **Data Availability** section below.***"**

- Line 117: "performed an additional (experiment) with samples..."

We have corrected this typo in the revised manuscript:

"*We, therefore, performed an additional experiment with samples taken over a shorter 2 hour 40 min period for a subset of the recombinases (****Figure 2b****, black crosses).***"**

- Figure 3c is not mentioned in the main text.

We apologise for this omission and have included a link to the figure panel in the revision:

"*Analysis showed a clear propagation of inversions in the coding sequences of the recombinases and GFP over time that corresponded to the sequential expression of each recombinase in the cascade (****Figure 3c****).***"**

Response to reviewers (NCOMMS-25-52996A)

Find below our response (in blue) to all the reviewers' comments. We believe these address the final minor comments made.

Reviewer #1:

I am satisfied with the improvements, and I thank the authors for acting on my comments. I have no outstanding comments and recommend publication.

We thank the reviewer for their efforts and are pleased that they are happy with the changes we made.

Reviewer #2:

The authors have addressed my concerns and the manuscript has been substantially improved. I have one minor comment regarding lines 240–249. The discussion appropriately identifies several factors that may slow recombination. If flipping is slow, unconverted plasmids may persist and continue to replicate during the experimental period, potentially limiting the extent of conversion. Thus, the observed dynamics may depend on the relative rates of recombinase-mediated flipping and plasmid replication or dilution, rather than on the flipping rate alone. Based on the green curve in Fig. 4d, these processes appear to occur on broadly similar timescales.

This is an excellent point. We have updated this section to include the additional insight: "*Furthermore, if the flipping of a segment is slow, unconverted plasmids may persist and continue to replicate as the experiment proceeds, resulting in a stable unflipped population of plasmids. Therefore, the dynamics we observe is also likely to depend on the relative rates of recombinase-mediated flipping and plasmid replication and dilution. This may account for the non-complete flipping of the gfp segment in the experiments (**Figure 4d**), a result of flipping, replication and dilution occurring on similar timescales.***"**
