# Supplementary material for: Characterization of recombinase-based genetic parts and circuits using nanopore sequencing
Source: Nat Commun. 2026 Sep 18;17:10969. doi: 10.1038/s41467-026-77605-x (PMC13683369; doi:10.1038/s41467-026-77605-x)
Supplement: Supplementary file 7 [file 41467_2026_77605_MOESM5_ESM.zip › integrase_model.pdf]

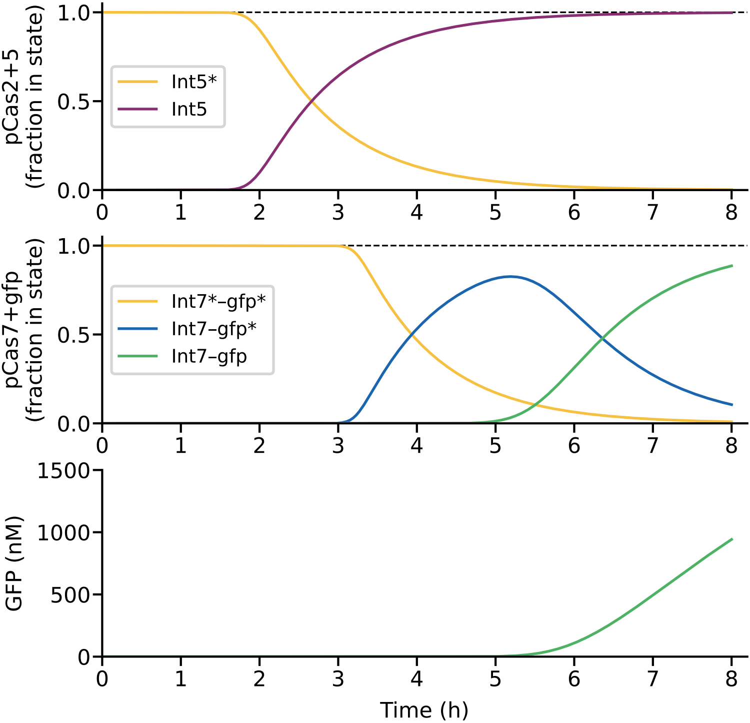

pCas2+5
(fraction in state)
1.0
0.5
0.0
Int5*
Int5
0
1
2
3
4
5
6
7
8
pCas7+gfp
(fraction in state)
1.0
0.5
0.0
Int7*–gfp*
Int7–gfp*
Int7–gfp
0
1
2
3
4
5
6
7
8
GFP (nM)
1500
1000
500
0
0
1
2
3
4
5
6
7
8
Time (h)
